# Cellular Responses of *Candida albicans* to Phagocytosis and the Extracellular Activities of Neutrophils Are Critical to Counteract Carbohydrate Starvation, Oxidative and Nitrosative Stress

**DOI:** 10.1371/journal.pone.0052850

**Published:** 2012-12-21

**Authors:** Pedro Miramón, Christine Dunker, Hanna Windecker, Iryna M. Bohovych, Alistair J. P. Brown, Oliver Kurzai, Bernhard Hube

**Affiliations:** 1 Department of Microbial Pathogenicity Mechanisms, Leibniz Institute for Natural Product Research and Infection Biology, Hans-Knoell-Institute, Jena, Germany; 2 Septomics Research Centre, Friedrich Schiller University and Leibniz Institute for Natural Products Research and Infection Biology, Hans-Knoell-Institute, Jena, Germany; 3 Aberdeen Fungal Group, Institute of Medical Sciences, University of Aberdeen, Foresterhill, Aberdeen, United Kingdom; 4 Center for Sepsis Control and Care, Universitätsklinikum Jena, Jena, Germany; 5 Friedrich Schiller University, Jena, Germany; Instituto de Salud Carlos III, Spain

## Abstract

Neutrophils are key players during *Candida albicans* infection. However, the relative contributions of neutrophil activities to fungal clearance and the relative importance of the fungal responses that counteract these activities remain unclear. We studied the contributions of the intra- and extracellular antifungal activities of human neutrophils using diagnostic Green Fluorescent Protein (GFP)-marked *C. albicans* strains. We found that a carbohydrate starvation response, as indicated by up-regulation of glyoxylate cycle genes, was only induced upon phagocytosis of the fungus. Similarly, the nitrosative stress response was only observed in internalised fungal cells. In contrast, the response to oxidative stress was observed in both phagocytosed and non-phagocytosed fungal cells, indicating that oxidative stress is imposed both intra- and extracellularly. We assessed the contributions of carbohydrate starvation, oxidative and nitrosative stress as antifungal activities by analysing the resistance to neutrophil killing of *C. albicans* mutants lacking key glyoxylate cycle, oxidative and nitrosative stress genes. We found that the glyoxylate cycle plays a crucial role in fungal resistance against neutrophils. The inability to respond to oxidative stress (in cells lacking superoxide dismutase 5 or glutathione reductase 2) renders *C. albicans* susceptible to neutrophil killing, due to the accumulation of reactive oxygen species (ROS). We also show that neutrophil-derived nitric oxide is crucial for the killing of *C. albicans*: a *yhb1*Δ/Δ mutant, unable to detoxify NO^•^, was more susceptible to neutrophils, and this phenotype was rescued by the nitric oxide scavenger carboxy-PTIO. The stress responses of *C. albicans* to neutrophils are partially regulated via the stress regulator Hog1 since a *hog1*Δ/Δ mutant was clearly less resistant to neutrophils and unable to respond properly to neutrophil-derived attack. Our data indicate that an appropriate fungal response to all three antifungal activities, carbohydrate starvation, nitrosative stress and oxidative stress, is essential for full wild type resistance to neutrophils.

## Introduction

*Candida albicans* is a polymorphic opportunistic fungus, commonly found as a commensal in healthy subjects [1]. It colonises distinct niches in the human body such as the oral and vaginal cavities and gut. *C. albicans* causes superficial infections, including oral and vaginal thrush, and deep-seated life-threatening infections such as disseminated candidiasis.

One of the best investigated virulence attributes of *C. albicans* is the ability to switch between yeast and hyphal growth forms. Both morphologies seem to be important for virulence and have distinct functions during the different stages of disease progression, including adhesion, invasion, damage, dissemination, immune evasion and host response [2]. The balance between host factors and virulence attributes of *C. albicans* determines the outcome of the interaction leading to commensalism or infection [3]. However, certain predisposing factor increase the risk of candidiasis, for instance, long-term antibiotic therapy, immunosuppression or disruption of anatomical barriers. Not surprisingly, the host is able to sense and respond to *C. albicans*. In the commensal state, *C. albicans* is tolerated, for example by the oral or vaginal epithelium, to prevent an inflammatory reaction against a relatively harmless component of the microbiota. However, epithelial cells are able to sense when this fungus represents a danger, because of increased proliferation or hyphal production, thereby inducing a proinflammatory response [4]–[6].

The response of the host towards the potentially dangerous *C. albicans* includes the recruitment of phagocytes from blood and tissues, considered as the first line of defence. Monocytes and granulocytes are immune cells with phagocytic activity that circulate in the bloodstream, priming the endothelium and responding to inflammatory signals coming from infected tissues. Neutrophilic granulocytes are unique in the response towards pathogens, since they respond rapidly and aggressively to potentially dangerous microorganisms. Neutrophils employ diverse antimicrobial killing mechanisms that include intra- and extracellular activities, as well as oxidative and non-oxidative mechanisms [7]. Neutrophils express receptors on their surface, including TLR2, TLR4 and dectin-1, that mediate the recognition of fungal cells, while other receptors, such as FCγR and CR3, aid in the opsonin-dependent phagocytosis of microorganisms [8].

Upon phagocytosis, the phagosome fuses with preformed granules containing several enzymes (e. g. cathepsin G, neutrophil elastase) and antimicrobial cationic peptides (α-defensins). A notable characteristic of the neutrophils is the production of copious amounts of oxidants in a process known as the respiratory burst [9]. It involves the assembly of the enzyme complex NADPH oxidase, on the plasma and phagosomal membranes of the phagocyte. This enzyme complex produces the highly reactive superoxide anion (O_2_^−•^), which is further metabolised to form hydrogen peroxide (H_2_O_2_). Other reactive species are produced inside the neutrophil. For instance, peroxynitrite (ONOO^−^) is formed upon production of nitric oxide (NO^•^) by the inducible nitric oxide synthase (iNOS) [10]. These two compounds are collectively known as reactive nitrogen species, and are extremely toxic for cells, reacting with thiol groups in proteins, thereby inactivating them. Additional compounds with oxidative properties (for example, hypochlorous acid, HClO), are produced by myeloperoxidase, an enzyme that is highly abundant in the neutrophil granules [7]. Degranulation is the hallmark of all neutrophil activities. It involves the secretion of peptides and enzymes stored in the neutrophil granules. Amongst the granule components, myeloperoxidase, lactoferrin and azurocidin are known to have candidacidal properties [11]–[13]. The production of neutrophil extracellular traps (NETs) provides another mechanism by which neutrophils contain infection. This involves the extrusion of chromatin scaffolding net-like structures decorated with antimicrobial proteins [14].

During adaptation to the host, *C. albicans* has evolved the ability to respond to host-generated stresses. We have shown that of all the cellular types present in blood, neutrophilic granulocytes exert the strongest effect on *C. albicans*, by inhibiting growth and evoking a response at the transcriptional level to overcome stresses such as carbohydrate and nitrogen starvation and oxidative stress, which contributes to the relatively high resistance of *C. albicans* to neutrophils *in vitro* [15]. It has long been known that the glyoxylate cycle contributes to the virulence of *C. albicans* [16], and that genes encoding the enzymes of this cycle, e. g. *ICL1* and *MLS1*, are induced upon phagocytosis by murine macrophages [17] and human neutrophils [15]. This metabolic pathway enables the fungus to utilise two-carbon molecules as a carbon source, and the induction of this pathway probably reflects the nutrient-deprived environment inside the phagocytes. Additionally, *C. albicans* faces amino acid deprivation upon neutrophil phagocytosis, and upregulates pathways for the synthesis of methionine and arginine [18]. We have observed an up-regulation of ammonium permease coding genes (*MEP1* and *MEP2*), as well as genes encoding vacuolar proteases (*APR1*, *PRB1*, *PRB2*, *PRC1*), which might be involved in protein turnover under starvation conditions, supporting the idea of nutrient limitation, and in particular nitrogen starvation [19]. Furthermore, it has been shown that *C. albicans* cell surface-associated superoxide dismutases are expressed in response to murine derived phagocytes, and are necessary for resistance to macrophages since in the absence of these enzymes there is accumulation of toxic oxygen radicals that eventually increases the susceptibility of *C. albicans* to killing by these cells [20]. We have demonstrated that *SOD5* gene expression, which is normally activated during hyphal growth, is induced during interactions with neutrophils [15], despite of the repression of filamentation by these phagocytes. Although it has long been known that neutrophils are able to produce nitric oxide during bacterial infections [21], the contribution of this compound to fungal killing by neutrophils has not being studied. *C. albicans* is able to respond to nitric oxide by up-regulating genes such as *YHB1*, a nitric oxide dioxygenase, and *SSU1*, a sulphite transporter protein [22]. Although these authors suggest that NO^•^ production is not a major determinant of virulence, we and others [23] argue that nitric oxide is relevant as a killing mechanism during the interaction of *C. albicans* with neutrophils.

The response of *C. albicans* cells to nutrient deprivation and oxidative stress within blood is primarily caused by exposure to neutrophils [15]. However, it is not clear whether phagocytosis is essential for these transcriptional responses, because this type of study averages the molecular behaviour of the fungal cell population as a whole, only a portion of which is phagocytosed when the transcriptional profiles are monitored [15]. The starvation and stress responses could have arisen from a subpopulation of phagocytosed cells. Alternatively, non-phagocytosed cells that have made contact with neutrophils may also face nutrient deprivation. Therefore, in this study we assess the contributions of intra- and extracellular neutrophil activities towards the induction of the *C. albicans* responses associated with neutrophil exposure and investigate the importance of the up-regulated genes and key stress regulators for survival during the interaction of *C. albicans* with neutrophils.

## Results

### Validation of GFP reporters as markers of stress specific response

We used diagnostic GFP reporter strains to monitor *C. albicans* gene expression at a cellular level (“single cell profiling”). First, we characterised the *in vitro* expression pattern of each reporter used in this study to define their specificity for given stresses. The stressors were based on previous studies [24]–[26]. To induce carbohydrate starvation and the expression of glyoxylate cycle genes, the non-fermentable carbon source acetate (2% potassium acetate) was chosen. To impose oxidative stress, we used a moderate concentration of hydrogen peroxide (2 mM H_2_O_2_), and to expose the fungal cells to nitrosative stress, we used the nitric oxide-releasing compound S-nitrosoglutathione (GSNO, 0.6 mM).

**Figure 1:**
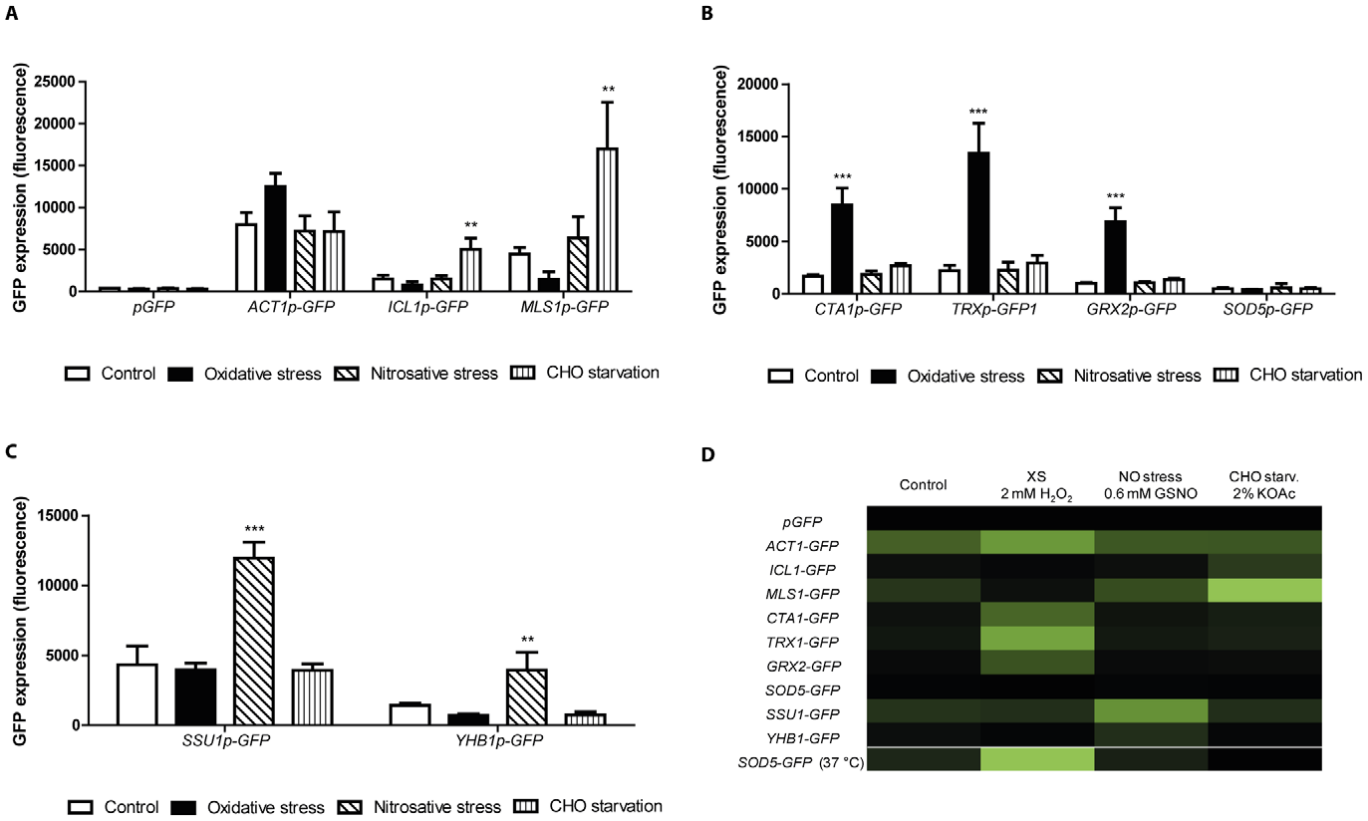
GFP reporter strains respond to oxidative stress, nitrosative stress and carbohydrate starvation. Exponential cultures of the GFP strains were exposed to oxidative stress (2 mM H_2_O_2_), nitrosative stress (0.6 mM GSNO) and carbohydrate starvation (2% potassium acetate). As controls, cultures were diluted in YPD medium. Fluorescence was quantified by FACS. (A) *pGFP* and *ACT1p-GFP* strains were used as controls. *ICL1p-GFP* and *MLS1p-GFP* showed increased fluorescence values under carbohydrate starvation. (B) Oxidative stress markers *CTA1p-*, *TRX1p-* and *GRX2p-GFP* showed increased GFP expression in the presence of H_2_O_2_. (C) Nitrosative stress reporters *YHB1p-* and *SSU1p-GFP* exhibited increased GFP fluorescence in the presence of the NO-releasing compound GSNO. (D) Heat map indicating the fluorescence intensity of each GFP reporter strain under the indicated stress (light green indicate highest expression). *SOD5p-GFP* increased the GFP fluorescence during exposure to oxidative stress (2 mM H_2_O_2_) only at 37°C. The average of three biological replicates is shown. **P≤0.01; ***P≤0.001, compared to the control.

*C. albicans* cells carrying *pGFP*, a promotorless GFP gene [27], were used as negative control. As shown in Fig. 1A, this strain exhibited no significant GFP fluorescence in all the conditions examined. In contrast, the positive control was the *C. albicans* strain carrying *ACT1p-GFP*, in which the GFP under the control of the actin gene (*ACT1*) promoter. *ACT1p-GFP* cells generally showed high fluorescence levels.

The *ICL1p-* and *MLS1p-GFP* reporters expressed GFP under the control of the promoters from the isocitrate lyase (*ICL1*) and malate synthase genes (*MLS1*) respectively. *C. albicans* cells with these reporters showed increased GFP fluorescence when grown on acetate as sole carbon source, a condition known to induce the glyoxylate cycle [16]. Both of these reporters displayed similar expression patterns, showing a 3.3–fold and 3.8–fold increase in fluorescent, respectively, during growth on acetate compared to glucose. Carbohydrate starvation was the only condition in which these reporter strains showed an increase in fluorescence relative to the control condition. However, they did display a decrease in fluorescence the presence of H_2_O_2_. No significant changes were observed under nitric oxide stress (Fig. 1A).

The oxidative stress reporters responded specifically to the presence of hydrogen peroxide (Fig. 1B). The *CTA1p-GFP* strain, expressing GFP under control of the catalase *CTA1* promoter, exhibited a 5-fold higher fluorescence intensity after hydrogen peroxide treatment compared to the unstressed control. Both, the *TRX1p-GFP* and *GRX2p-GFP* strains, in which GFP is under control of the thioredoxin *TRX1* and glutathione reductase *GRX2* promoters, respectively, displayed a >6-fold increase in fluorescence intensity when compared to the unstressed control (Fig. 1B). As expected, these oxidative stress-specific reporters showed no major changes in GFP fluorescence after carbohydrate starvation or in the presence of GSNO. Interestingly, the *SOD5p-GFP* cells, expressing GFP under control of the superoxide dismutase *SOD5* promoter, did not show any significant increase in fluorescence under any of the conditions tested at 30°C, regardless of the presence of H_2_O_2_. Therefore, we tested the expression of this GFP reporter under further conditions including increased temperature. At 37°C, the *SOD5p-GFP* strain responded to the presence of H_2_O_2_ (Fig. 1D). Under all other stress conditions, the GFP signal remained as low as in the unstressed control, thus indicating that *SOD5* is specifically up-regulated in response to oxidative stress at 37°C. No morphological changes were observed during the course of these experiments.

Finally, GFP expression levels were examined in the *SSU1p-* and *YHB1p-GFP* strains, which express GFP under the control of the sulphite transporter *SSU1* and nitric oxide dioxygenase *YHB1* promoters, respectively. As shown in Fig. 1C, the greatest GFP expression was observed when these strains were exposed to GSNO. Fluorescence intensities from these strains were not significantly changed in the unstressed control, under oxidative stress or under carbohydrate starvation. This indicates that the nitrosative stress reporters respond specifically to the presence of the nitric oxide-releasing compound GSNO.

Taken together, these results demonstrate that the GFP reporter strains respond to the relevant stress (Fig. 1D).

### Single cell profiling of the GFP reporters during coincubation with neutrophils

Having established that the GFP reporter strains respond specifically to the relevant stress conditions, we then investigated the responses of these reporters in the presence of neutrophils. Two subsets of *C. albicans* cells were analysed in a series of experiments: phagocytosed and non-phagocytosed cells. The latter set represented those cells that are attached to neutrophils without being internalised as well as cells that are not in direct contact with the phagocytes. To discriminate between internalised and non-internalised fungal cells, samples were counterstained using ConA-AF647, which binds strongly to non-phagocytosed *C. albicans* cells, while those within the neutrophils are faintly stained or not stained at all. As a control, each reporter strain was incubated under the same conditions in the absence of neutrophils.

**Figure 2:**
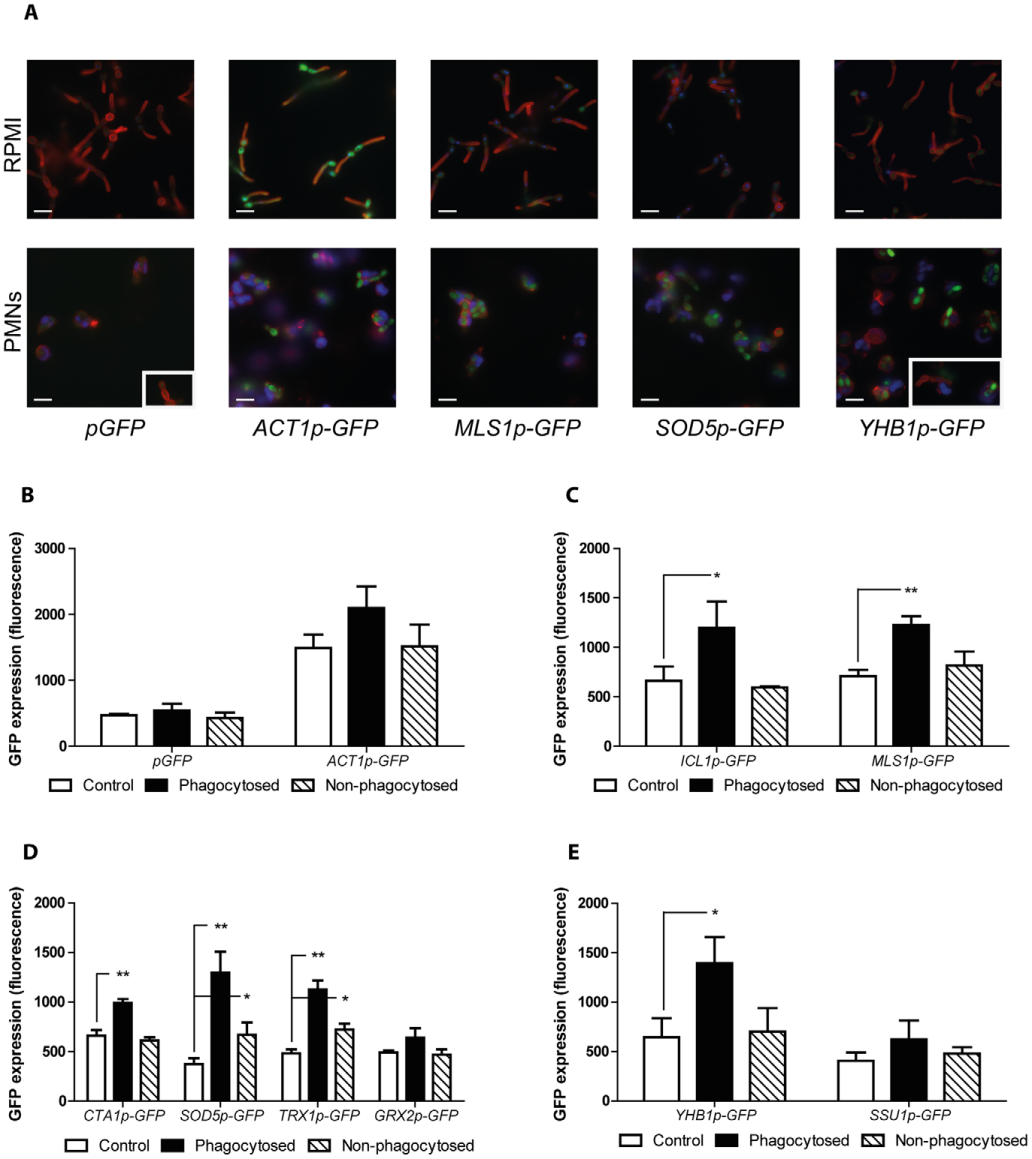
Expression analysis using GFP reporter strains during interaction with neutrophils. Exponential GFP reporter cells were co incubated with neutrophils for 1 h. The GFP signal was quantified as described in the experimental procedures. (A) Representative microphotographs of selected GFP strains incubated in RPMI +5% FBS in the absence of neutrophils or coincubated with the phagocytes. Boxes in the right lower corner belong to different fields of the same microphotograph. White bars represent 10 µm. (B) *pGFP* strains was used as negative control, and *ACT1p-GFP* as positive control. (C) The carbohydrate starvation reporters *ICL1p-* and *MLS1p-GFP* showed an increase in the GFP expression in those cells that were phagocytosed by the neutrophils, while the GFP expression in the non-phagocytosed cells remain as low as in the control without neutrophils. (D) Cells from the *CTA1p-* and *GRX2p-GFP* strains showed increased GFP expression in the phagocytosed population. The *SOD5p-* and *TRX1p-GFP* cells inside the neutrophils showed the highest fluorescent signal, however, cells that were not phagocytosed also had a significant increase in the GFP expression. (E) Reporter strains for the nitrosative stress response *YHB1p-* and *SSU1p-GFP* showed increased GFP expression only in those cells that were phagocytosed. The average of at three biological replicates is shown. *P≤0.05, **P≤0.01, compared to the control.

As shown in Fig. 2A and B, a low fluorescent signal was detected whenever the *pGFP* strain was used. In contrast, the *ACT1p-GFP* strain showed high fluorescence levels under all three conditions. The moderately higher fluorescence in phagocytosed cells was not statistically significant.

To determine whether carbohydrate starvation is imposed intra-, extracellularly or under both conditions, the *ICL1p-GFP* and *MLS1p-GFP* strains were used. These strains exhibited a higher fluorescence intensity only in the phagocytosed population (Fig. 2A and C), suggesting that carbohydrate starvation is imposed only in the intracellular environment.

For the oxidative stress reporters (*CTAp-*, *SOD5p*-, *TRX1p*- and *GRX2p-GFP*), the highest fluorescent signal was also observed in the phagocytosed fungal cells (Fig. 2D). However, some of these reporters also showed relatively high GFP expression levels before internalisation by the phagocytes (Fig. 2D). *CTA1p-GFP* exhibited the highest fluorescence when *C. albicans* cells were inside the neutrophils. *SOD5p*- and *TRX1p-GFP* reporter strains displayed similar expression patterns in that phagocytosed cells responded to the greatest extent. However, *SOD5p-* and *TRX1p-GFP* cells that remained outside of phagocytes also displayed significantly elevated GFP expression levels, indicating that they sensed and responded to extracellular neutrophil-derived reactive oxygen species. However, the full response occurred only following phagocytosis. Consistent with our previous results and despite the fact that *SOD5* expression is associated with the morphogenetic program [28], the fungal cells expressed the *SOD5p-GFP* construct even though they remained in the yeast morphology (data not shown). The *GRX2p-GFP* reporter also exhibited a slight increase in fluorescence in the phagocytosed population, but this increase was not statistically significant when compared to the control without neutrophils. Taken together, these results indicate that the full exposure and response to oxidants occurs in the intracellular environment, but some oxidative stress genes are induced by extracellular neutrophil-derived activities.

Next, we tested whether *C. albicans* readily responds to nitrosative stress. For this, we made use of the nitrosative stress reporter strains carrying *YHB1p-* and *SSU1p-GFP*. Both genes have been shown to be upregulated in response to nitric oxide [22]. As shown in Fig. 2A and E, these GFP reporters responded to neutrophils by increasing the fluorescence levels upon phagocytosis. *YHB1p-GFP* showed the strongest GFP induction, while up-regulation of *SSU1p-GFP* was evident, but not significant. We concluded that nitrosative stress is imposed only in the intracellular environment as *YHB1p-GFP* responded only following phagocytosis.

### The glyoxylate cycle but not gluconeogenesis is needed to resist neutrophil-mediated killing

**Figure 3:**
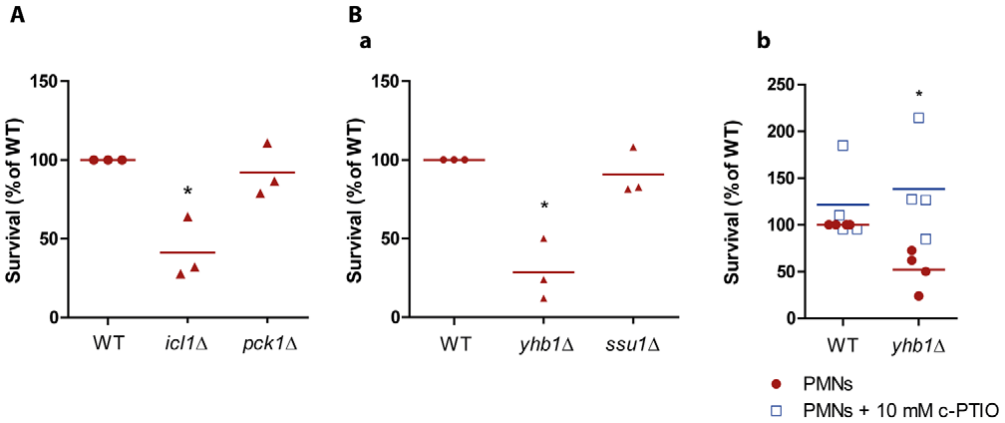
*C. albicans* mutants *icl1*Δ and *yhb1*Δ are more sensitive to neutrophil killing. (A) The mutant *icl1*Δ/Δ, lacking isocitrate lyase, a key enzyme involved in the glyoxylate cycle, is unable to resist the neutrophil killing as the wild type strain (RM1000 + CIp20). In contrast, a mutant defective in the phosphoenolpyruvate carboxykinase gene *PCK1* and unable to perform gluconeogenesis, showed no increased susceptibility. (B) a. The mutant *yhb1*Δ/Δ, lacking the nitric oxide dioxygenase 1, showed increased sensitivity to neutrophil killing than the parental wild type (BH117). A mutant lacking the gene *SSU1*, a gene coding for a sulphite transporter protein that is upregulated in response to nitric oxide, showed no increased sensitivity to neutrophil-mediated killing. Fungal cells were exposed to neutrophils for 3 h. Results from 3 independent replicates are shown. Survival of respective wild type strains was set to 100%. b. Survival assay using the nitric oxide scavenger carboxy-PTIO. Mutant *yhb1*Δ/Δ was exposed to human neutrophils in the presence of 10 mM carboxy-PTIO, under the standard conditions. Residual metabolic activity of the cells was determined as described. Survival of the wild type strain without carboxy-PTIO was set as 100%. Experiments were performed three independent times. *P≤0.05, compared to the control.

**Figure 4:**
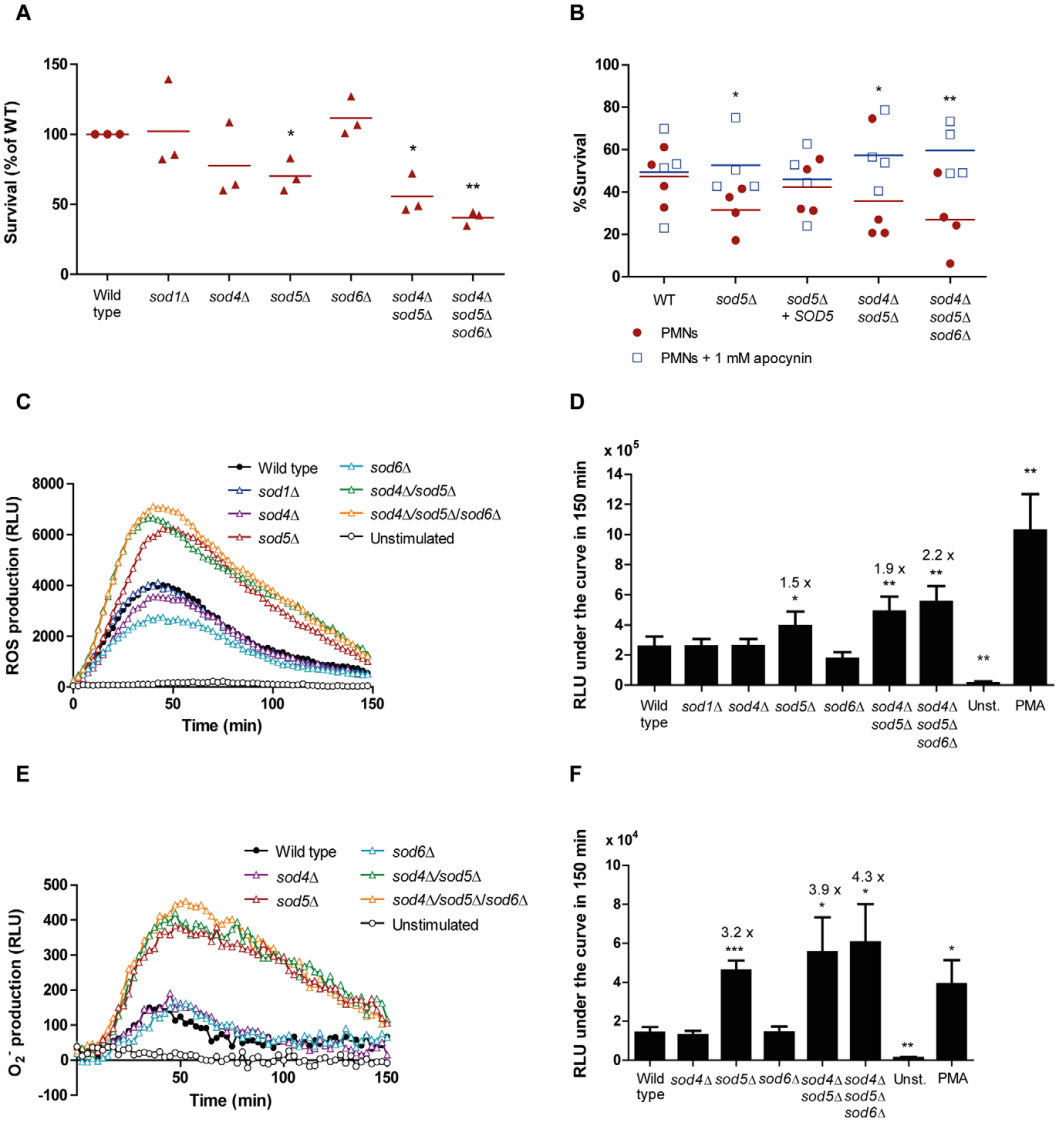
*C. albicans* mutants lacking *SOD5* are hypersensitive to neutrophil killing and induce ROS accumulation. (A) *C. albicans* mutants *sod5*Δ/Δ, *sod4/5* Δ/Δ and *sod4/5/6* Δ/Δ were more susceptible to neutrophil-mediated killing. Fungal cells were exposed to neutrophils for 3 h. Results from three independent replicates are shown, lines represent the mean value from the replicates. Survival of wild type strain was set to 100%. (B) Neutrophils were incubated with 1 mM apocynin for 30 min at 37°C with 5% CO_2_, before infection with opsonised fungal cells. Lines represent the mean values of each strain in the presence of neutrophils (filled red circles) and apocynin-treated neutrophils (open blue squares). Results from four replicates are shown. Statistical significance was tested by two-way ANOVA with Bonferroni post-tests. (C) Luminol-enhanced chemiluminescence assay was performed to determine the accumulation of neutrophil-derived ROS upon stimulation with *C. albicans* mutants (MOI 1) lacking genes coding for superoxide dismutases. Neutrophils were left unstimulated or stimulated with 20 nM PMA (not shown) as negative and positive controls, respectively. A representative result of one replicate is shown. (D) The area under the curve was calculated to determine the total ROS accumulation during the course of the experiment (150 min). The average of four independent replicates is presented. (E) Oxidative burst assay using lucigenin to specifically detect the superoxide (O_2_^−^) radical production upon neutrophils stimulation with *C. albicans* mutants (MOI 1). Neutrophils were left unstimulated or stimulated with 20 nM PMA (not shown) as negative and positive controls, respectively. A representative result of one replicate is shown. (F) The area under the curve was calculated to determine the total superoxide accumulation during the course of the experiment (150 min). The average of four independent replicates is shown. *P≤0.05; **P≤0.01; compared to the control.

**Figure 5:**
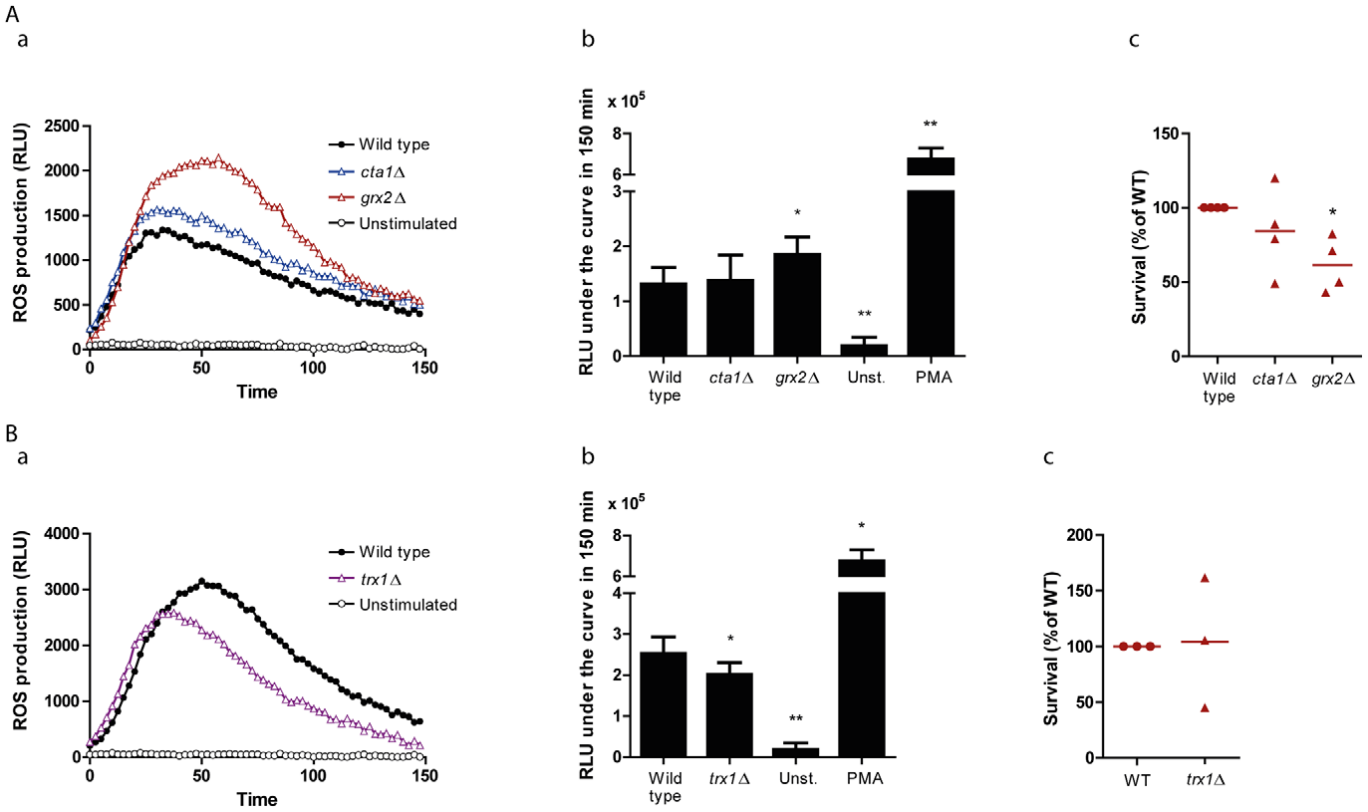
ROS induction and survival of deletion mutants *cta1*Δ/Δ, *grx2*Δ/Δ and *trx1*Δ/Δ. (A) a. Upon neutrophil confrontation with strain *grx2*Δ/Δ, ROS accumulated. Deletion mutant lacking the catalase coding gene, *cta1*Δ/Δ, had only a slight and not significant increase in ROS accumulation, when compared to the wild type strain (CAI4 + CIp10). A representative result of one replicate is shown. b. Quantification of the area under the curve. Stimulation of neutrophils with *grx2*Δ/Δ but not with *cta1*Δ/Δ led to significant accumulation of ROS. Unstimulated and PMA-stimulated neutrophils were used as negative and positive controls, respectively. The average of three independent experiments is presented. c. *C. albicans* mutant *grx2*Δ/Δ is reduced in the wild type survival capacity when exposed to neutrophils. In contrast, *cta1*Δ/Δ did not show a significant decrease in the sensitivity to neutrophils. Fungal cells were exposed to neutrophils for 3 h. Results from four independent replicates are shown. Survival of the wild type strain was set to 100%. (B) a. ROS production in response to *trx1*Δ/Δ. Neutrophil-derived ROS are slightly decreased upon stimulation with a *trx1*Δ/Δ mutant, when compared to the isogenic WT strain (SN148 + CIp30). b. Quantification of the area under the curve. Data represent the average of three independent experiments. c. The sensitivity of the *trx1*Δ/Δ mutant towards neutrophil killing was not clearly affected as compared to the WT. Results from three independent replicates are shown. Survival of the WT was set to 100%. *P≤0.05; **P≤0.01; compared to the control.

Having characterised reporter gene activation during *C. albicans*-neutrophil interactions, the contribution of the corresponding gene products to fungal fitness and survival was evaluated. To achieve this we determined the susceptibility of the mutants in a neutrophil-mediated killing assay (Fig. 3–5). In this assay, fungal cells lacking the specific factors were confronted with freshly isolated human neutrophils. The susceptibility of each mutant strain was then normalised against the susceptibility of an isogenic wild type strain.

Based on previously performed transcriptional analyses, it was known that *C. albicans* up-regulates the glyoxylate cycle when confronted to neutrophils, most likely to enable the fungus to use alternative carbon sources in a glucose-deprived environment. We hypothesised that the inability to metabolise via the glyoxylate cycle would render *C. albicans* more susceptible to neutrophil-mediated killing. As predicted, an *icl1*Δ/Δ mutant lacking the isocitrate lyase gene showed a significant increase in susceptibility to neutrophil killing (Fig. 3A). We also predicted that glyconeogenesis, a metabolic pathway required for the generation of hexoses in a sugar-scarce environment, might also contribute to fitness. However, when we examined a *pck1*Δ/Δ mutant, which lacks phosphoenolpyruvate carboxykinase and is unable to perform gluconeogenesis, this strain showed no significant increase in the susceptibility to neutrophil killing (Fig. 3A). This suggested that this pathway is not essential for survival under these conditions. This is in accordance to the transcriptional data, where the genes coding for the key enzymes of this pathway, *FBP1* and *PCK1*, were not up-regulated in the presence of neutrophils [15].

### Superoxide dismutase 5 (Sod5) is pivotal for normal resistance to neutrophil-derived ROS

Next, we investigated whether superoxide dismutases are crucial to counteract the oxidative stress imposed by neutrophils. *C. albicans* possesses an isoenzyme family of six superoxide dismutases localised in different cellular compartments. Superoxide dismutases 1, 2 and 3 (Sod1-3) are intracellular enzymes, whereas Sod4, 5 and 6 are GPI-anchored cell wall-associated enzymes that face the extracellular environment. *SOD5* expression is induced in response to neutrophils and a mutant defective in *SOD5* is unable to resist neutrophil killing at wild type levels [15]. Moreover, Sod5 has been linked to the detoxification of ROS derived from myeloid dendritic cells and bone marrow-derived macrophages [20]. As shown in the expression analysis, *SOD5* was upregulated during *C. albicans*-neutrophil interactions. The expression of this gene is normally associated with filamentation, but expression was increased in fungal cells that did not undergo the morphogenetic transition once internalised by neutrophils [15]. The *sod5*Δ/Δ mutant was clearly more susceptible to neutrophil killing than the respective wild type (P value  = 0.0474, Fig. 4A). To assess the contribution of the additional surface-associated Sods, we tested single *sod4*Δ/Δ and *sod6*Δ/Δ mutants, a double *sod4*Δ/Δ *sod5*Δ/Δ mutant and a triple *sod4*Δ/Δ *sod5*Δ/Δ *sod6*Δ/Δ mutant in the neutrophil killing assay, as well as the *sod1*Δ/Δ mutant, which lacks the cytoplasmic Sod1. As shown in Fig. 4A, the *sod1*Δ/Δ mutant was as sensitive to neutrophil killing as the wild type strain, suggesting that Sod1 does not play a major role in the detoxification of ROS generated by neutrophils. Neither the single *sod4*Δ/Δ mutant nor the *sod6*Δ/Δ mutant displayed significant changes in sensitivity to neutrophils, compared to the wild type control. In contrast, the double *sod4*Δ/Δ *sod5*Δ/Δ (P value  = 0.0326) and the triple *sod4*Δ/Δ *sod5*Δ/Δ *sod6*Δ/Δ (P value  = 0.0024) mutants were only slightly more sensitive than the *sod5*Δ/Δ single mutant, suggesting that, despite the presence of apparently redundant Sods, Sod5 plays the pivotal role in the detoxification of neutrophil-derived oxidative stress.

Next, we tested whether inhibition of the neutrophil NADPH oxidase complex rescues the hypersensitive phenotype of *sod5*Δ/Δ cells, since superoxide production in neutrophils relies almost exclusively on this enzymatic complex. The inhibition of NADPH oxidase with apocynin completely abolished superoxide production (data not shown). After challenging apocynin-treated neutrophils with *C. albicans*, the *sod5*Δ/Δ mutant displayed similar survival rates to wild type cells (Fig. 4B). Furthermore, the survival of the double and triple *sod* mutants increased to the wild type levels when challenged with apocynin-treated neutrophils. Interestingly, almost no effect could be observed in the survival of the wild type strain upon NADPH oxidase inhibition.

To determine whether there is a correlation between the generation and accumulation of ROS and increased susceptibility to neutrophil killing, we measured the production and accumulation of neutrophil-derived ROS. As shown in Fig. 4C–D, neutrophils exposed to *sod1*Δ/Δ, *sod4*Δ/Δ and *sod6*Δ/Δ mutants produced ROS at comparable levels to those produced upon challenge with the wild type strain. In stark contrast, *sod5*Δ/Δ, *sod4*Δ/Δ *sod5*Δ/Δ and *sod4*Δ/Δ *sod5*Δ/Δ *sod6*Δ/Δ caused more ROS accumulation. To determine whether superoxide is accumulated upon stimulation with the *sod5*Δ/Δ mutants, a luminescent determination using lucigenin as the chemiluminescent substrate, which specifically detects the superoxide anion, was performed. As shown in Fig. 4E–F, more than 3-fold more superoxide accumulated upon neutrophil challenge with *sod5*Δ/Δ, *sod4*Δ/Δ *sod5*Δ/Δ or *sod4*Δ/Δ *sod5*Δ/Δ *sod6*Δ/Δ cells compared with wild type cells. In contrast, exposure to *sod4*Δ/Δ or *sod6*Δ/Δ cells did not cause increased superoxide accumulation. This suggests that, following exposure to neutrophils, Sod5 is the major enzyme required for ROS detoxification and in particular for superoxide detoxification. No significant differences in superoxide accumulation were detected between the single *sod5*Δ/Δ mutant and the double *sod4*Δ/Δ *sod5*Δ/Δ and triple *sod4*Δ/Δ *sod5*Δ/Δ *sod6*Δ/Δ mutants, although a trend of increased accumulation was observed.

We next investigated whether the elevated ROS accumulation was due to increased activation of the neutrophils by these mutants. We quantified the expression of the activation markers CD11b (also known as integrin α_M_) and CD66b (also known as CEACAM8) on the surface of the phagocytes. Neutrophils stimulated with any of the *sod*Δ/Δ mutants were activated to the same extent as the wild type strain, as indicated by the expression of these two activation markers on the surface of the neutrophils (Fig. S1 A–B). This suggested that the increased ROS accumulation was mediated by the reduced ability of the *C. albicans* cells to eliminate the oxidants rather than by increased neutrophil activation.

### Mutants defective in the catalase activity do not exhibit significantly increased sensitivity towards neutrophil-mediated killing

Next, we tested mutants lacking further key effectors of the oxidative stress response. It has been previously shown that several genes encoding enzymes or signalling pathway components involved in the oxidative stress response in *C. albicans* are up-regulated during the interaction with neutrophils [15]. Moreover, we demonstrated the expression of some of these genes (*CTA1*, *TRX1*, *GRX2*) in phagocytosed and non-phagocytosed fungal cells by using GFP reporter strains (Fig. 2). We hypothesised that mutants lacking the corresponding genes might display increased susceptibility to neutrophils and that the ROS levels in neutrophils exposed to these mutants would be increased due to inefficient detoxification. First, we investigated ROS levels in neutrophils exposed to a *cta1*Δ/Δ mutant, lacking detectable catalase activity (Alistair J. P. Brown, unpublished data). Interestingly, despite the lack of catalase activity, this mutant induced ROS almost to the same extent as the wild type (Fig. 5A a–b). Moreover, when we determined the residual metabolic activity of the *cta1*Δ/Δ mutant after confrontation with neutrophils, we found that although this mutant showed a trend towards decreased survival, the susceptibility in the killing assay was not significantly increased (Fig. 5A). This suggested that, even though *CTA1* is upregulated and may be involved in the detoxification of neutrophil-derived oxidants, specifically H_2_O_2_, the catalase activity might be dispensable for normal susceptibility to neutrophils.

### Glutathione reductase activity is crucial for ROS detoxification and resistance to neutrophils

We also investigated a mutant lacking the gene coding for the glutathione reductase *GRX2*, involved in the reduction of oxidised glutathione, a tripeptide associated with the oxidative stress response. As shown in Fig. 5A a, ROS accumulated when the *grx2*Δ/Δ mutant was coincubated with neutrophils in the oxidative burst assay, and this accumulation was significantly greater than for neutrophils coincubated with wild type *C. albicans* cells (Fig. 5A b). The total amount of ROS accumulated with *grx2*Δ/Δ cells was not as striking for the *sod5*Δ/Δ and related mutants, and this might be related to the inability of these *C. albicans* cells to fully detoxify the oxidants generated upon neutrophil activation. Consistent with the increased accumulation of ROS, the *grx2*Δ/Δ mutant exhibited a decreased survival after it was confronted with neutrophils for 3 h (Fig. 5A c).

Since we showed that *grx2*Δ/Δ also induces accumulation of ROS at significant levels, we investigated whether superoxide was one of the accumulated ROS. In contrast to the *sod5*Δ/Δ mutants, the *grx2*Δ/Δ did not induce accumulation of superoxide (Fig. S2 B–C), suggesting that this mutant is not defective in the ability to eliminate this radical, but probably unable to keep the redox balance necessary for the maintenance of reduced glutathione in the cell after oxidative stress exposure. Strikingly though, other ROS-independent mechanisms may be rendering this mutant more susceptible, since inhibition of the NADPH oxidase, did not have an effect on the survival outcome (Fig. S2 A).

We then investigated the role of thioredoxin, which is involved in oxidative stress sensing and detoxification [29]. In the presence of neutrophils, TRX1-GFP levels were markedly up-regulated (Fig. 2C). The behaviour of the *trx1*Δ/Δ mutant in the oxidative burst assay was different to the other mutants, since it consistently induced slightly less ROS production than its isogenic wild type control (Fig. 5B a–b). Moreover, we did not observe a clear effect upon the survival of this mutant upon exposure to neutrophils in the killing assay (Fig. 5B c).

### Inability to detoxify neutrophil-derived RNS renders C. albicans more susceptible to killing

The next aspect we focused on was the ability of *C. albicans* to overcome neutrophil-derived nitrosative stress. In our expression analyses, we showed that *C. albicans* up-regulates the *YHB1* gene. Furthermore, *SSU1*, a gene which expression is induced in the presence of nitric oxide, was also up-regulated in response to neutrophil phagocytosis. We then tested a mutant lacking the *YHB1* gene and determined the susceptibility of it when confronted with neutrophils. Consistent with our observation, the mutant *yhb1*Δ/Δ showed a clear increase in sensitivity to neutrophil killing (Fig. 3B a), suggesting that nitric oxide detoxification contributes to the survival of *C. albicans* following neutrophil-derived nitrosative stress. To test whether the neutrophil sensitivity of *yhb1*Δ/Δ cells is related to their defect in nitric oxide detoxification, we tested whether the neutrophil sensitivity of *yhb1*Δ/Δ cells can be suppressed by the nitric oxide scavenger carboxy-PTIO. Consistent with our hypothesis, the survival of *yhb1*Δ/Δ cells was significantly improved in the presence of the nitric oxide scavenger (Fig. 3B b), providing evidence that nitrosative stress plays a significant role in the killing of *C. albicans* by neutrophils. *SSU1* encodes a sulphite transport protein consistently expressed upon nitric oxide exposure [22]. In our assay, the *ssu1*Δ/Δ mutant showed no change in the susceptibility to neutrophils (Fig. 3B a). The role of sulphite mobilisation and the nitric oxide metabolism in *C. albicans* remains ambiguous. Our data suggest that Ssu1 is not required for nitric oxide detoxification, although *SSU1* is up-regulated in response to nitric oxide and neutrophils.

### The stress regulator Hog1 is required for normal survival of C. albicans upon neutrophil attack

**Figure 6:**
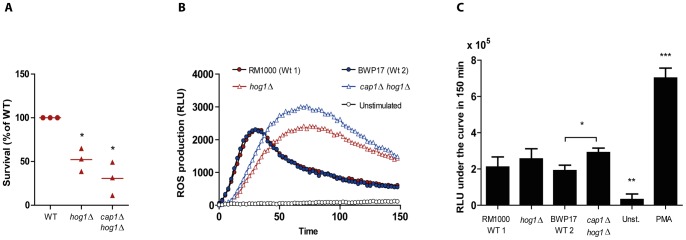
Behaviour of mutants lacking the stress regulator Hog1 during the interaction with neutrophils. (A) *C. albicans* mutants *hog1*Δ/Δ and *hog1*Δ/Δ *cap1*Δ/Δ were more sensitive to killing by neutrophils. Fungal cells were exposed to neutrophils for 3 h. Results from three independent replicates are shown. Survival of wild type strains was set to 100%. (B) The mutant *hog1*Δ/Δ lacking the stress regulator Hog1 induced the generation of neutrophil-derived ROS with a clear delay as compared to the respective wild type (RM1000 + CIp20). This delayed induction of ROS was also observed when neutrophils were challenged with a double *hog1*Δ/Δ *cap1*Δ/Δ mutant, lacking the oxidative stress regulator Cap1 in addition to Hog1. For the double mutant, the accumulation of ROS was statistically significant, compared to the wild type (BWP17 + CIp30). Unstimulated and PMA-stimulated (not shown) neutrophils were included as negative and positive controls, respectively. A representative result of one replicate is shown. (C) Determination of the area under the curve from the ROS measurements. The single mutant *hog1*Δ/Δ accumulated only slightly more ROS than the WT during the course of the experiments. Likewise, the double mutant *hog1*Δ/Δ *cap1*Δ/Δ caused more ROS accumulation, upon coincubation with neutrophils. The average of three independent replicates is shown. *P≤0.05; **P≤0.01; ***P≤0.001, compared to the control.

Stress responses in *C. albicans* are largely coordinated by the stress regulator Hog1, a stress activated protein kinase that mediates the expression of genes in response to oxidative, osmotic and heavy metal stress [30]. In addition to an increased sensitivity to oxidative stress, the *hog1*Δ/Δ mutant displays morphological defects, forming filaments under non-inducing conditions [31], [32]. We hypothesised that the inability to mount a robust stress response, in addition to the morphogenetic defects, will render the *hog1*Δ/Δ mutant hypersensitive to killing by neutrophils. When confronted to neutrophils, *hog1*Δ/Δ was clearly impaired in its ability to resist phagocytic attack (Fig. 6A). This decreased resistance to neutrophils was even more dramatic in the double mutant *hog1*Δ/Δ *cap1*Δ/Δ, which also lacks the gene encoding Cap1, a regulator that mediates the transcriptional response to oxidative stress.

To provide some mechanistic explanation for this increased sensitivity to neutrophils, we investigated the behaviour of these mutants in the oxidative burst assay. When neutrophils were exposed to *hog1*Δ/Δ cells, the kinetics of ROS production differed significantly compared to the parental wild type strain, showing a clear delay (Fig. 6B). When the total ROS accumulation was determined, *hog1*Δ/Δ showed only slightly and not significantly increased ROS levels as compared to the wild type *C. albicans* control, despite the altered dynamics. Similarly, the double mutant *hog1*Δ/Δ *cap1*Δ/Δ also induced a delayed ROS production response. In addition, the total ROS induced by this double mutant was significantly higher in comparison to the single *hog1*Δ/Δ mutant (Fig. 6C).

To test whether the increased ROS production was due to increased neutrophil activation by these mutants, we assayed the expression of the activation markers CD11b and CD66b on the neutrophil surface. CD11b expression was not significantly altered in response to the single mutant *hog1*Δ/Δ or the double mutant *hog1*Δ/Δ *cap1*Δ/Δ compared to their respective wild type strains (Fig. S1 C–D). However, we detected a moderate but significantly lower expression of CD66b upon stimulation of neutrophils with *hog1*Δ/Δ cells after 1 h co-incubation (Fig. S1 D).

Taken together, these results suggest that the inability of these mutants to properly respond to the neutrophil-generated oxidative stress renders them significantly more sensitive to the attack by these phagocytes, and highlights the relevance of Hog1 signalling during the interactions between fungal and innate immune cells.

## Discussion

Neutrophils are one of the most important types of phagocytes during *Candida* infections, acting as a first line of defence. We have previously shown that, amongst blood cell types, neutrophils exert the greatest effects upon *C. albicans* and govern the transcriptional response in blood infections [15]. Upon attack by neutrophils, *C. albicans* activates specific responses that include the up-regulation of metabolic pathways necessary for the assimilation of alternative carbon sources, and the induction of proteins involved in the detoxification of oxidants. Moreover, the up-regulation of genes that normally respond to the presence of nitric oxide led us to hypothesise that nitrosative stress is a relevant stress that *C. albicans* encounters during interaction with neutrophils.

Neutrophils can kill microorganisms either by phagocytosis (a process involving the sensing, recognition and ingestion of microorganisms) or by extracellular mechanisms involving the release of antimicrobial compounds, such as oxidants, enzymes or peptides. Using a single cell profiling approach, we show that carbohydrate starvation is imposed only upon fungal cells that have undergone phagocytosis. The genes coding for the glyoxylate cycle enzymes isocitrate lyase (*ICL1*) and malate synthase (*MLS1*) were up-regulated upon phagocytosis. These findings are in accordance to Barelle et al. [24] and suggest that phagocytosed fungal cells are exposed to an intracellular environment that is relatively poor in carbohydrates. Therefore, in order to survive, *C. albicans* is forced to use alternative carbon sources that might be available within the phagocyte (such as organic acids). Alternatively, *C. albicans* might refocus its metabolism such that it can recycle its own carbon-containing molecules. The relevance of the glyoxylate cycle in the survival of *C. albicans* upon exposure to neutrophils is supported by the finding that an *icl1*Δ/Δ mutant showed decreased resistance to neutrophil-mediated damage, similar to observations made with macrophages [16]. Under the same conditions one would expect an additional up-regulation of genes involved in gluconeogenesis, since this pathway drives the synthesis of hexoses under starvation conditions. However, our previous study suggested that this pathway is not up-regulated during the interaction with neutrophils [15]. In addition, it has been shown that *PCK1*, encoding a central enzyme of gluconeogenesis, is not up-regulated upon phagocytosis by neutrophils [24], and we provide evidence in this study that the inability to perform gluconeogenesis has no effect in the overall survival of *C. albicans* after exposure to these phagocytes (Fig. 3A). The data suggest that the glyoxylate cycle, but not gluconeogenesis, plays a key role in the adaptive responses of phagocytosed *C. albicans* cells.

*C. albicans* responds robustly to oxidative insults. The fungus possesses a number of signalling pathways that regulate several mechanisms to cope with the oxidative stress [33]. In agreement with the “commensal school” concept [34], it is not surprising that this otherwise commensal organism has evolved the ability to cope with oxidative stress. The fungus probably encounters phagocytes, at least transiently, during its normal commensal life style, which utilise oxidants to damage and kill microorganisms. *C. albicans* is able to detoxify the superoxide anion, which is produced by the enzyme complex NADPH oxidase, present in all types of phagocytes. To accomplish this, *C. albicans* has a family of superoxide dismutases that transform superoxide into hydrogen peroxide. The main focus of research regarding *C. albicans* Sods has been the surface-associated Sods, namely Sod4 and Sod5, because they are responsible for the immediate ROS detoxification upon exposure to superoxide. It has been shown that these enzymes display morphotype-specific expression patterns: yeast cells readily express Sod4, while expression of Sod5 is detected under hypha-inducing conditions [28], [35]. We have demonstrated that *C. albicans* up-regulates the *SOD5* gene, not only in response to phagocytosis by neutrophils, but also to extracellular oxidants produced by the phagocytes, since fungal cells that remain outside of neutrophils display significantly elevated *SOD5* expression levels. Moreover, *C. albicans* relies on Sod5 to resist the neutrophil attack, since *sod5*Δ/Δ mutants are more sensitive to neutrophil killing. We provide evidence supporting the idea that superoxide detoxification contributes significantly to the normal outcome in the survival of this pathogen after confrontation with neutrophils. In the absence of Sod5, neutrophil-derived ROS accumulated upon stimulation. This is in accordance with previous work where it was shown that ROS accumulated upon stimulation of bone-marrow derived macrophages (BMDMs) and myeloid dendritic cells (mDCs) from mice with mutants lacking Sod5 [20]. Our results demonstrate that superoxide is accumulated as part of the total ROS. The lack of *SOD5* significantly contributes to the ROS accumulation. This was further supported by the observation that specific inhibition of the NADPH oxidase by apocynin greatly enhanced the survival of *sod5*Δ/Δ mutants. Similarly, other fungal pathogens also rely on the ability to detoxify host-derived ROS via superoxide dismutases. For example, it has recently been shown that *Histoplasma capsulatum* requires Sod3, a surface-associated superoxide dismutase, to successfully overcome the killing by murine cytokine-stimulated macrophages and human neutrophils, and that Sod3 is directly involved in the elimination of host-derived ROS [36].

Neutrophil-derived superoxide anion is further transformed into hydrogen peroxide. Amongst the enzymes capable of catabolising hydrogen peroxide are catalase and peroxidases. *C. albicans* possesses one gene encoding catalase, *CTA1*. We and others [25] have shown that this gene is induced upon phagocytosis, whereas extracellular neutrophil activities did not seem to influence the expression of this gene. When confronted with neutrophils, the *cta1*Δ/Δ mutant did not show a significant reduction in its ability to survive the phagocyte attack although we observed a trend towards decreased survival. This finding is in contrast to earlier work [37] where a *cta1*Δ/Δ mutant was tested and shown to be significantly reduced in survival when exposed to neutrophils at different MOIs, including the MOI used in this study. However, in that previous work, the authors tested the susceptibility of 3 h-induced hyphae in RPMI at 37°C, while in our study the infection was done with cells of the yeast morphotype, which eventually formed germ tubes during the course of the experiment. This may explain the difference in the two studies and may suggest a different role of Cta1 in yeast and hyphal cells. Surprisingly, the *cta1*Δ/Δ mutant did not significantly induce the accumulation of total ROS. Since this mutant is unable to metabolise H_2_O_2_ via catalase, this oxidant was expected to accumulate, and therefore be detected in the ROS assay. This finding indicates that the catalase activity is dispensable for normal resistance to neutrophils, despite the fact that *CTA1p-GFP* expression was strongly induced upon neutrophil phagocytosis. It is therefore possible that *C. albicans* relies on other activities to further metabolise and detoxify critical and detrimental H_2_O_2_ levels. For example, *C. albicans* up-regulates genes coding for peroxidases when coincubated with neutrophils [15]. Whether peroxidase activity is responsible for the detoxification of H_2_O_2_ in the absence of catalase activity remains unclear. Another hydrogen peroxide detoxification mechanism potentially used by *C. albicans* when confronted with neutrophils or other phagocytes could be the induction of glutathione as an oxidant scavenger. Glutathione is a small molecule that possesses two thiol groups that are sensitive to oxidation by H_2_O_2_. Once oxidised, glutathione can be reduced and thus recycled by the action of glutathione reductase, using NADPH as electron donor. *C. albicans* has at least two genes coding for putative glutathione reductases, *GRX2* and *GLR1*. Both genes were shown to be up-regulated in the presence of neutrophils [15]. This finding is in accordance with another previous study, which showed that this gene was up-regulated in the presence of neutrophils, but not by macrophages [25]. In our study, we showed that *GRX2* (also known as *TTR1*) was moderately up-regulated upon phagocytosis by neutrophils. In agreement with Chaves et al. [38] we found that a *grx2*Δ/Δ mutant was hypersensitive to neutrophils, which is likely to contribute to the observed attenuated virulence of the mutant in a murine model of disseminated candidiasis. One explanation for the increased sensitivity of this mutant to neutrophil killing might be its inability to readily detoxify neutrophil-derived oxidants other than superoxide (Fig. 5A a), possibly H_2_O_2_, since this mutant did not accumulate the superoxide radical (Fig. S2). However, since we observed no increase in the survival of this mutant upon inhibition of the NADPH oxidase, the most relevant source of oxidants in the neutrophil, it is also possible that other non-oxidative mechanisms are responsible for the increased susceptibility of this mutant.

In addition to detoxification mechanisms, *C. albicans* possesses proteins involved in sensing the presence of oxidants. Thioredoxin, encoded by *TRX1*, has recently been shown to be involved in sensing of oxidative stress [29]. Supporting this observation, our data show that, *TRX1* is up-regulated during co-incubation with neutrophils. This up-regulation occurs after phagocytosis, but also in cells that have not been ingested. However, the sensitivity of a *trx1*Δ/Δ mutant to neutrophils was not increased. Consistently, accumulation of ROS was not increased, but significantly decreased in this mutant when challenged with neutrophils. We conclude that, despite the involvement of Trx1 in the oxidative stress sensing mechanisms, Trx1 is dispensable for survival after neutrophil exposure. The fact that this mutant was not more sensitive to neutrophil killing, but still displayed attenuated virulence in mice [29] shows that Trx1 may have additional roles *in vivo* or simply that the role of Trx1 during the interaction with neutrophils *in vivo* cannot be mimicked in the *in vitro* setting. Since the *trx1*Δ/Δ mutant has morphological defects [29], it may also be possible that these defects contribute to reduced recognition, reduced stimulation of neutrophils and/or reduced virulence of this mutant. Phagocytes are well known for their ability to generate ROS, but they are also capable of producing nitric oxide and related metabolites, altogether referred as reactive nitrogen species (RNS). The production of nitric oxide by phagocytes largely depends on the inducible nitric oxide synthase, or iNOS. We provide evidence that *C. albicans* responds to neutrophil-derived nitric oxide, and that this neutrophil activity contributes to the killing of *C. albicans*, demonstrating that nitric oxide detoxification has a significant impact on the survival of this fungus in presence of neutrophils. We confirmed an observation of a previous study from our group that the gene *YHB1*, coding for a nitric oxide dioxygenase, is induced during co-incubation with neutrophils [15], using a *YHB1p*-GFP reporter strain, which responded to the presence of the nitric oxide-releasing compound GSNO. We further showed that the *YHB1* expression occurred only upon phagocytosis, indicating that its expression contributes to fungal extracellular defence mechanisms against neutrophils. This is in accordance with the dynamics of the generation of nitric oxide by the phagocytes. Whereas superoxide is produced by the active NADPH oxidase complex assembled in cytoplasmic and phagosomal membranes, the nitric oxide synthase is localised in the cytoplasm or associated with vesicles that fuse with the phagosome, delivering the nitric oxide directly into this organelle [10]. Thus, the nitric oxide is only encountered in the intracellular environment. Moreover, lack of *YHB1* rendered *C. albicans* clearly more susceptible to neutrophil-mediated killing, highlighting the importance of nitric oxide detoxification. The ability to cope with nitric oxide is advantageous for pathogens to successfully colonise the host. Several examples are found in bacteria, noteworthy *Escherichia coli* [39], *Salmonella* serovar Typhimurium [40] and *Mycobacterium tuberculosis* [41]. These bacterial pathogens possess flavohaemoglobins that detoxify host-generated nitric oxide, providing protection against macrophage-derived nitric oxide and thus improving their survival when confronted with phagocytes. In the case of fungal pathogens, the contribution of host-derived nitrosative stress is less well studied. However, an earlier report showed that *Cryptococcus neoformans* relies on two nitric oxide detoxification systems, the flavohaemoglobin Fhb1, that consumes nitric oxide, and a GSNO reductase Gno1, that metabolises GSNO [42]. Mutants lacking these enzymes showed attenuated virulence in a mouse model. Similarly, as a consequence of the inability to detoxify nitric oxide, *C. albicans yhb1*Δ/Δ mutants showed reduced virulence in models of disseminated candidiasis [22], [23], [43]. The fact that *C. albicans yhb1*Δ/Δ cells were protected against neutrophils in the presence of carboxy-PTIO, a nitric oxide scavenger, suggests that the underlying reason for the increased sensitivity of this mutant to neutrophil killing is linked to the nitric oxide exposure upon attack by the phagocytes. It seems unlikely that neutrophil-derived nitric oxide is sufficient to directly kill *C. albicans*, but it is possible that nitric oxide exerts its effect by halting the fungal growth [44], and acts synergistically with other fungicidal mechanisms, like oxidants, antimicrobial peptides or lytic enzymes. In addition, nitric oxide by-products (peroxynitrite, peroxynitrous acid and others) may participate in the damage and eventual killing of the fungus.

The ability of *C. albicans* to sense and respond to stress largely depends on the Hog1 signalling pathway. It has been shown that mutants lacking Hog1 have an increased susceptibility to host immune cells [26] and an overall decreased virulence in a model of disseminated candidiasis [45]. We included the *hog1*Δ/Δ mutant in our study in order to evaluate the role of stress response signalling. Consistent with previous works, lack of *HOG1* renders *C. albicans* more sensitive to the killing by neutrophils. Interestingly, the *hog1*Δ/Δ mutant exhibited a clear delay in the induction of neutrophil-derived ROS. Despite this delayed induction, it accumulated slightly more ROS than the respective wild type. This was also observed in the double mutant *hog1*Δ/Δ *cap1*Δ/Δ, lacking an additional key regulator of stress response signalling, in a more pronounced manner, and the accumulation of ROS by this double mutant was actually significantly higher. The reasons underlying the delayed ROS induction, and therefore neutrophil stimulation, may rely on the morphological defects of the *hog1*Δ/Δ mutants [31], [32], which exhibits hyphal formation even under non-inducing conditions. In fact, the morphology of *C. albicans* plays a crucial role in neutrophil activation and mutants with morphological defects are unable to activate the neutrophil ERK1-kinase, involved in motility and killing of *C. albicans* [46]. We initially hypothesised that, if the *hog1*Δ/Δ mutant exhibits a hyperfilamentous phenotype, it may greatly induced the production of ROS. However, as shown by Wozniok et al. [46], the filamentous morphotype exhibited by some mutants is not sufficient to properly activate the neutrophils. This is also supported by the observation that the expression of the activation markers CD11b and Cd66b on neutrophils upon stimulation with either the *hog1*Δ/Δ or the *hog1*Δ/Δ *cap1*Δ/Δ mutants was not increased as compared to the wild type. In addition, it has been documented that deletion of *HOG1* has a profound effect on the cell wall architecture, which is associated with a constitutive activation of the Cek1 cell wall-stress pathway [45], which in turn may also account for the differences we observed in the dynamics of the ROS induction. The overall outcome of the lack of Hog1 either in the *hog1*Δ/Δ or the *hog1*Δ/Δ *cap1*Δ/Δ mutants is a decreased survival. We have previously shown that the *cap1*Δ/Δ mutant is more sensitive to neutrophil killing [15]. The fact that the double mutant is more prone to killing evidences that both stress signalling pathways are necessary for full wild type resistance of *C. albicans* to neutrophils.

Taken together, our results show that *C. albicans* responds to a combination of insults upon neutrophil attack, including nutritional, oxidative and nitrosative stresses. It shows that some of these stresses are imposed exclusively in the intracellular milieu, like carbohydrate starvation or exposure to nitric oxide, while others occur in both the intra- and extracellular environments, such as insults of oxidative nature. In addition to starvation and oxidative stress, nitrosative stress is relevant for neutrophil-mediated killing of this fungus, and *C. albicans* responds readily to this kind of insult. Moreover, the integrity of signalling pathways such as Hog1 and Cap1 are pivotal for the normal resistance of this pathogen when confronted with neutrophils. We have dissected the responses at the transcriptional level using GFP reporter strains, and we have assessed the actual contribution of the gene products for the survival of *C. albicans* by using mutant strains. Although this works emphasises the relevance of certain stresses imposed by the neutrophil, it is expected that other mechanisms contribute to the killing of *C. albicans*, for instance, the production of non-oxidative molecules such as lytic enzymes (e. g. cathepsin G, neutrophil elastase), cationic peptides (α-defensins, LL-37, histones) or metal chelator proteins (lactoferrin, calprotectin).

## Experimental Procedures

### Ethics statement

Blood was obtained from healthy human donors with written informed consent. The blood donation protocol and use of blood for this study were approved by the institutional ethics committee (Ethik-Kommission des Universitätsklinikum Jena, Permission No 2207-01/08).

### Strains and culture conditions

Strains used in this work are listed in table S1. Strains were routinely grown in liquid YPD (1% yeast extract, 2% bacto-peptone, 2% D-glucose, 2% agar if needed), or SD minimal medium (2% D-glucose, 0.17% yeast nitrogen base, 0.5% ammonium sulphate, 2% agar if needed). For liquid overnight cultures, strains were grown in YPD in a shaking incubator at 30°C, 180 rpm. Exponential cultures were obtained by diluting an overnight culture in fresh YPD to an OD_600_ of 0.2 and incubated at 30°C and 180 rpm until the culture reached an OD_600_ of 0.5.

### Strain construction

*C. albicans* CAI-4 was used for the construction of the *MLS1p*-, *SSU1p*- and *YHB1p*-*GFP* reporter strains, in order to use the same genetic background as the previously described GFP strains [24], [25], [27]. Primers sequences are listed in table S2. To generate the plasmid pMLS1-GFP, the *C. albicans MLS1* promoter (−1000 to −1, relative to the start codon) was PCR amplified with the primers MLS1 Xho1 5′ and MLS1 HindIII 3′ and cloned between the XhoI and HindIII sites of pGFP [27]. To construct pSSU1-GFP, the *SSU1* promoter (−1000 to −1) was PCR amplified with the primers SSU1 XhoI 5′ and SSU1 PstI 3′, and cloned between the respective sites in pGFP. Likewise, to generate pYHB1-GFP, the *YHB1* promoter (−3077 to −1) was amplified with the primers YHB1 XhoI 5′ and YHB1 MluI 3′ and cloned between the XhoI and MluI sites in pGFP. Plasmids were linearised with StuI, integrated in the *RPS10* locus in CAI-4 and correct chromosomal integration was PCR confirmed as described elsewhere [27].

### GFP reporter stress – specific response

The GFP reporter strains were grown overnight in YPD, diluted in fresh YPD to an OD_600_ of 0.2 and regrown to an OD_600_ of 0.5. Carbohydrate starvation was imposed by diluting the cultures to an OD_600_ of 0.1 in 2% potassium acetate – YP medium. Oxidative stress was imposed by diluting the cultures in 2 mM H_2_O_2_ – YPD. Similarly, nitrosative stress was imposed by diluting the cultures in 0.6 mM S-nitrosoglutathione (GSNO, Sigma Aldrich) –YPD. As controls, strains were grown in YPD medium without stress. After 2 h of exposure at 30°C, samples were fixed with 4% paraformaldehyde, washed with 1x Dulbecco's Phosphate-Buffered Saline (DPBS, PAA) and analysed by fluorescence activated cell analysis (FACS, BD Biosciences, UK). The median GFP fluorescence from 10 000 events was recorded. Three independent experiments were performed.

### Isolation of polymorphonuclear cells from human peripheral blood

Neutrophils were isolated using a gradient centrifugation method. Briefly, 1 part of 1x DPBS-diluted blood was layered on top of 1 part of Polymorphprep (Axis-Shield) and centrifuged for 45 min at 500 g, at 20°C. After centrifugation, plasma and monocyte layer were discarded. The polymorphonuclear cell (PMN) fraction was collected in a fresh tube containing 1 volume of 0.5x DPBS and centrifuged for 10 min at 400 g. Residual erythrocytes were removed with ACK lysing buffer (Life Technologies). Neutrophils were washed with 1x DPBS and resuspended in RPMI1640+5% heat inactivated (10 min at 56°C) foetal bovine serum (FBS), if not stated otherwise.

### Single cell profiling using GFP reporters in an ex vivo neutrophil infection model

The response of GFP reporter strains to neutrophil phagocytosis or extracellular activities was examined using an *ex vivo* infection model and a counterstain to differentiate between phagocytosed and non-phagocytosed fungal cells. Freshly isolated neutrophils were seeded in wells with a 12 mm diameter gelatine – coated coverslip at the bottom, at a concentration of 2.5×10^6^ cells ml^−1^ in a volume of 400 µl. Neutrophils were incubated for 1 h at 37°C with 5% CO_2_ to let them attach to the glass surface. An aliquot of exponentially growing cultures of the GFP strains was washed twice with 1x DPBS. The cells were diluted to a concentration of 4×10^7^ fungal cells ml^−1^ in RPMI1640+5% FBS and 100 µl of this suspension were used to infect the neutrophils (MOI 2). Infection was allowed to proceed for 1 h, at 37°C with 5% CO_2_. Cells were fixed with 4% paraformaldehyde and carefully washed with 1x DPBS. Counterstaining was performed using 5 µg concanavalin A – Alexa Fluor 647 (ConA – AF647, Life Technologies) ml^−1^, which readily binds to non-phagocytosed cells and poorly, or not at all, to intracellular fungal cells. After washing with 1x DPBS, coverslips were mounted upside down on slides with ProLong Gold Antifade Reagent with DAPI (Life Technologies). Fluorescence microscopy was performed in a Leica DM5500B microscope, using appropriate filters for the detection of GFP, Alexa Fluor 647 and DAPI fluorescence. Micrographs were taken in the Leica Application Suite (Leica Microsystems, Mannheim). Fluorescence measurement was done using the quantification tool for regions of interest. For each reporter strain, the GFP fluorescence was determined in at least 30 phagocytosed and 30 non-phagocytosed fungal cells. As controls, the GFP reporters were incubated in wells without neutrophils. All strains were tested in three independent biological replicates.

### Susceptibility to neutrophil killing – XTT assay

To determine the susceptibility of different *C. albicans* deletion mutants, cells from exponential cultures were washed twice with 1x DPBS, and opsonised with 50% human serum (diluted in 1x DPBS), for 30 min at 37°C. Neutrophils (10^6^ cells ml^−1^) were infected with opsonised *C. albicans* (10^6^ cells ml^−1^) at a 1∶1 ratio, in 200 µl final volume. To inhibit the NADPH oxidase, neutrophils were pre-incubated with 1 mM apocynin (Sigma) for at least 30 min before infection with fungal cells. The nitric oxide scavenger carboxy-PTIO (Sigma) was used at a final concentration of 10 mM, when required. Cells were incubated for 3 h at 37°C and 5% CO_2_. Control samples with only fungal cells were incubated and further processed under the same conditions. Neutrophils were lysed by addition of 1 ml cold water. Fungal cells were centrifuged for 5 min at 16 000 g, at 4°C and 1 ml of the supernatant was carefully removed to avoid aspiration of the pellet. 400 µl of 0.5 mg ml^−1^ XTT (Sigma) and 50 µg ml^−1^ coenzyme Q_0_ (Sigma) diluted in 1x DPBS were added. Samples were incubated for 1 h at 37°C and centrifuged for 5 min at 16 000 g, at 4°C. Supernatants were used to measure the absorbance at 450 nm in a Tecan Infinite M200 microplate reader. Background absorbance (neutrophils only and medium only) was subtracted from the samples. The residual metabolic activity was used a measurement of the survival and it was calculated as the quotient of the absorbance of the coincubation (*C. albicans* strains and neutrophils) and the absorbance of the *C. albicans* strains alone, multiplied by 100. Survival of the isogenic wild type strain was set to 100%. Experiments were done at least three times, using neutrophils from different donors.

### Neutrophil activation

The activation of neutrophils upon stimulation with *C. albicans* deletion strains was determined by means of reactive oxygen species (ROS) production in an oxidative burst assay, and changes in the expression of receptors on the neutrophil surface.

The oxidative burst assay was performed using the luminol-enhanced chemiluminescence method for the quantification of total reactive oxygen species. For the specific detection of the superoxide anion, lucigenin was used as the chemiluminescent probe. To prevent unspecific activation of the neutrophils, wells from a 96-well white plate (Corning) were coated with 0.05% albumin (in 1x DPBS) for 1 h at 4°C, and washed twice with 1x DPBS afterwards. Freshly isolated neutrophils were resuspended in RPMI1640 w/o phenol red with 5% FBS at a concentration of 5×10^5^ cells ml^−1^ and 100 µl of this suspension were seeded. Neutrophils were incubated for 30 min to 1 h at 37°C and 5% CO_2_, to let them attach. Fungal cells from exponential cultures were washed and opsonised as described, and resuspended in RPMI1640 at a concentration of 10^6^ cells ml^−1^. Neutrophils were stimulated with 50 µl of the fungal suspensions. As positive control, neutrophils were stimulated with 20 nM phorbol 12-myristate 13-acetate (PMA, Sigma Aldrich). A control of unstimulated neutrophils was included in each experiment. A control consisting of each *C. albicans* strain only was included in every experiment, and the luminescence of this control was subtracted from the luminescence from the coincubation. Immediately after stimulation, 50 µl of RPMI1640 containing either 200 µM luminol (Sigma Aldrich) and 16 U HRP (Sigma Aldrich) to detect total ROS, or 400 µM lucigenin (Sigma Aldrich) to detect superoxide, were added and chemiluminescence was measured every 2.5 min for 2.5 h in a Tecan Infinite M200 microplate reader. The area under the curve was calculated in GraphPad Prism 5.03. Experiments were done in at least three independent occasions using neutrophils from different donors. RPMI1640 w/o phenol red was used to avoid interference with the chemiluminescence signal.

The detection of neutrophil surface receptors as markers of activation was done by means of FACS, using FITC-stained *C. albicans* cells, to ease the gating of neutrophils in association with fungal cells. Exponential cultures were washed and opsonised as described before. Staining was done using a 100 µg ml^−1^ FITC solution in 0.15 M NaCl 0.1 M Na_2_CO_3_ buffer, pH 9.0. Fungal cells were resuspended in this solution and incubated for 30 min at 30°C. Cells were centrifuged and thoroughly washed with 1x DPBS, before being resuspended and diluted in RPMI1640+5% FBS. 2×10^5^ neutrophils were stimulated with opsonised FITC-stained *C. albicans* cells at an MOI of 1 for 1 h or 1.5 h at 37°C in the presence of 5% CO_2_. Unstimulated neutrophils were used as negative control and neutrophils stimulated with 40 nM PMA were used as positive control. After incubation, cells were centrifuged at 400 g for 5 min at 4°C and washed with 1% BSA – 1x DPBS. Cells were resuspended and incubated with 500 ng anti CD66b – PE (BioLegend), 125 ng anti CD11b – Pacific Blue (BioLegend), human serum and 1% BSA – 1x DPBS for 20 min at 4°C in the dark. Cells were collected at 400 g for 5 min and resuspended in 500 µl cell wash. Analysis was done in FACSDiva (BD Bioscience, UK) and FlowJo. Neutrophils were gated in the dot plots as CD66b^+^, and the median fluorescence signal was determined in the FITC positive population, to determine the expression of the receptors in the neutrophils in contact with *C. albicans* cells.

### Statistical analysis

Data are presented as the mean of at least three independent biological replicates, error bars represent standard deviation. Statistical tests were performed using GraphPad Prism 5.03. Two-tailed Student's *t*-test was used for two groups comparison. When more than two groups were compared, one-way analysis of variance (ANOVA) with Tukey post-tests was used. Two-way ANOVA with Bonferroni post-tests was used to analyse the results from the survival assays upon NADPH inhibition, or nitric oxide scavenging.

## Supporting Information

Figure S1 **Neutrophil expression of CD11b and CD66b upon stimulation with** ***sod*** **Δ/Δ,** ***hog1*** **Δ/Δ and** ***hog1*** **Δ/Δ** ***cap1*** **Δ/Δ mutants.** Surface expression of the activation markers CD11b (A and C) and CD66b (B and D) on neutrophils stimulated with FITC-stained *C. albicans* (MOI 1), analysed by FACS. A and B show results after 1 h exposure to the *sod*Δ/Δ mutants. C and D show results of 1 h and 1.5 h exposure to *hog1*Δ/Δ and *hog1*Δ/Δ *cap1*Δ/Δ mutants. The average of the median fluorescence intensities of at least three biological replicates is shown. Data represent the fluorescence values from neutrophils associated with *C. albicans* cells (phagocytosed or attached). Unstimulated and PMA-stimulated neutrophils were used as negative and positive controls, respectively. **P≤0.01, compared to the control.
(TIF)

Figure S2 **Neutrophil-derived superoxide does not contribute to the hypersensitive phenotype of the mutant** ***grx2*** **Δ/Δ.** (A) Apocynin-treated neutrophils were infected with opsonised fungal cells. Infection was allowed to proceed for three hours and residual metabolic activity was determined at the end. Lines represent the mean values of each strain in the presence of neutrophils (filled red circles) and apocynin-treated neutrophils (open blue squares). Results from four replicates are shown. Statistical significance was tested by two-way ANOVA with Bonferroni post-tests. (B) Detection of superoxide radicals using lucigenin as chemiluminescent probe. One representative replicate is shown. Neutrophils were left unstimulated as negative control. (C) Quantification of area under the curve. Superoxide production from PMA-stimulated neutrophils was included as the positive control. Results from four independent replicates are shown.
(TIF)

Table S1 *C. albicans* strains used in this work.
(DOCX)

Table S2 Primers used in this work.
(DOCX)
