# Supplementary figures and images for: Cellular Responses of Candida albicans to Phagocytosis and the Extracellular Activities of Neutrophils Are Critical to Counteract Carbohydrate Starvation, Oxidative and Nitrosative Stress
Source: PLoS One. 2012 Dec 21;7(12):e52850. doi: 10.1371/journal.pone.0052850 (PMC3528649; doi:10.1371/journal.pone.0052850)

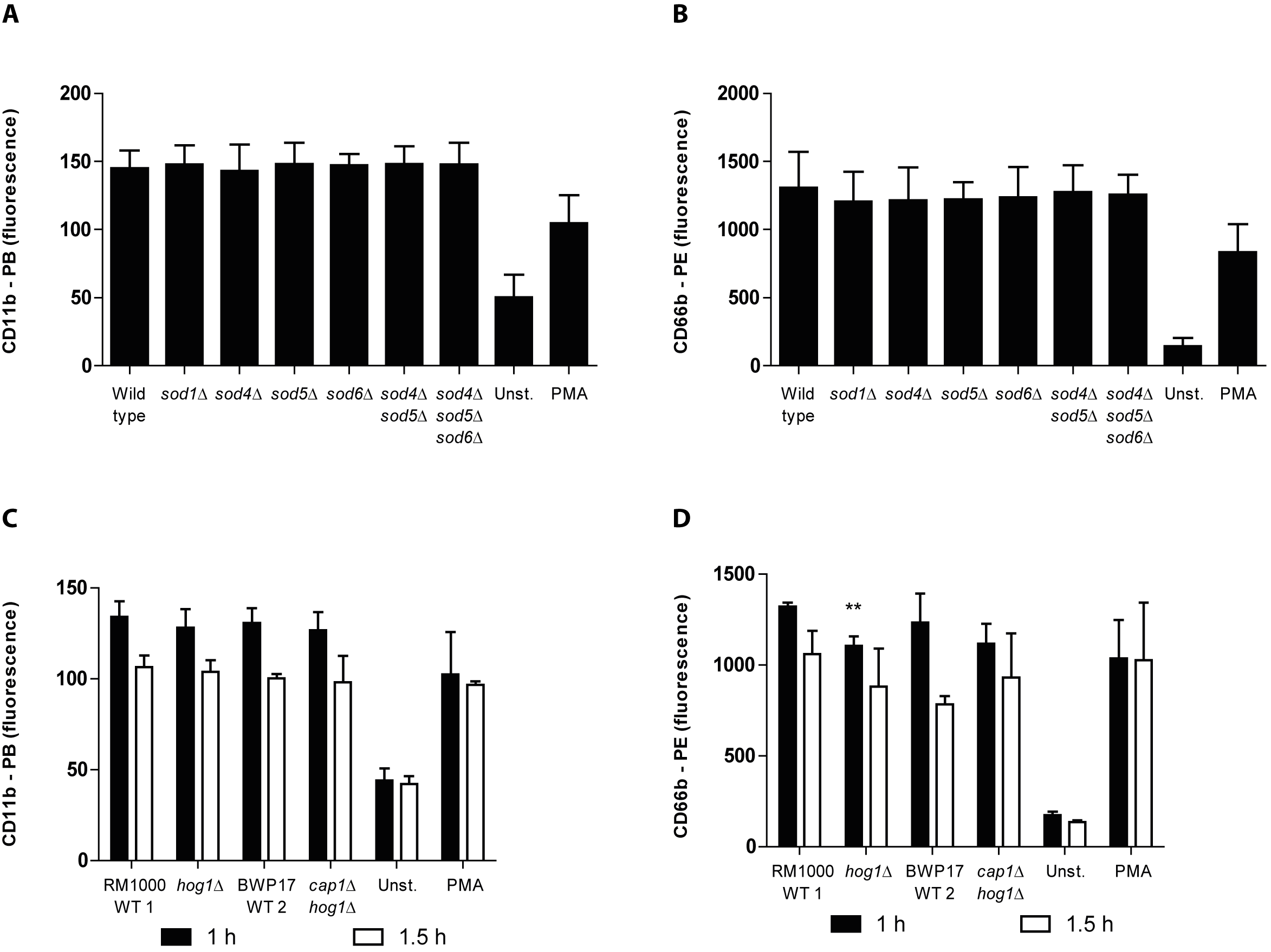

Supplement: Figure S1 — Neutrophil expression of CD11b and CD66b upon stimulation with sod Δ/Δ, hog1 Δ/Δ and hog1 Δ/Δ cap1 Δ/Δ mutants. Surface expression of the activation markers CD11b (A and C) and CD66b (B and D) on neutrophils stimulated with FITC-stained C. albicans (MOI 1), analysed by FACS. A and B show results after 1 h exposure to the sodΔ/Δ mutants. C and D show results of 1 h and 1.5 h exposure to hog1Δ/Δ and hog1Δ/Δ cap1Δ/Δ mutants. The average of the median fluorescence intensities of at least three biological replicates is shown. Data represent the fluorescence values from neutrophils associated with C. albicans cells (phagocytosed or attached). Unstimulated and PMA-stimulated neutrophils were used as negative and positive controls, respectively. **P≤0.01, compared to the control. (TIF) [file pone.0052850.s001.tif]

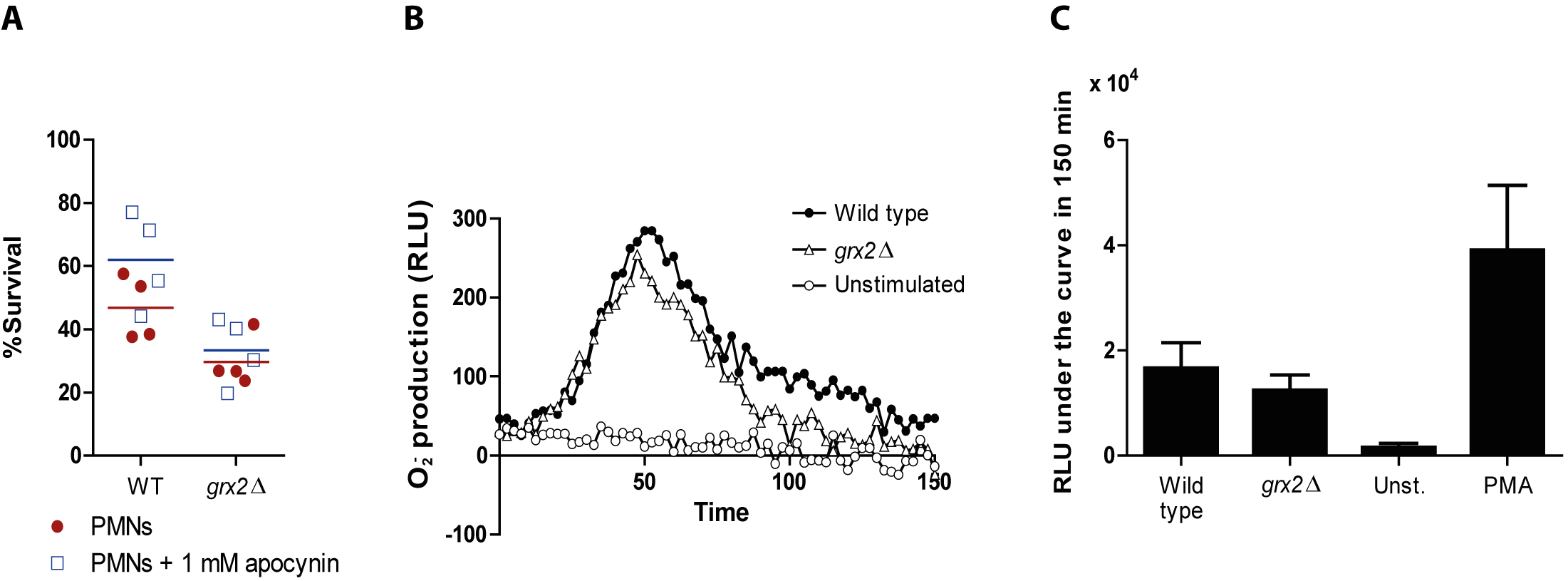

Supplement: Figure S2 — Neutrophil-derived superoxide does not contribute to the hypersensitive phenotype of the mutant grx2 Δ/Δ. (A) Apocynin-treated neutrophils were infected with opsonised fungal cells. Infection was allowed to proceed for three hours and residual metabolic activity was determined at the end. Lines represent the mean values of each strain in the presence of neutrophils (filled red circles) and apocynin-treated neutrophils (open blue squares). Results from four replicates are shown. Statistical significance was tested by two-way ANOVA with Bonferroni post-tests. (B) Detection of superoxide radicals using lucigenin as chemiluminescent probe. One representative replicate is shown. Neutrophils were left unstimulated as negative control. (C) Quantification of area under the curve. Superoxide production from PMA-stimulated neutrophils was included as the positive control. Results from four independent replicates are shown. (TIF) [file pone.0052850.s002.tif]
