# Supplementary material for: Cellular Responses of Candida albicans to Phagocytosis and the Extracellular Activities of Neutrophils Are Critical to Counteract Carbohydrate Starvation, Oxidative and Nitrosative Stress
Source: PLoS One. 2012 Dec 21;7(12):e52850. doi: 10.1371/journal.pone.0052850 (PMC3528649; doi:10.1371/journal.pone.0052850)
Supplement: Table S1 — C. albicans strains used in this work. (DOCX) [file pone.0052850.s003.docx]

**Table S1.** *C. albicans* strains used in this work.

| **Strain** | **Genotype** | **Function** | **Reference** |
| --- | --- | --- | --- |
| SC5314 | Clinical isolate | Wild type | [[1](#_ENREF_1)] |
| BWP17 | *ura3Δ::λimm434/ura3Δ::λimm434 arg4Δ::hisG/arg4Δ::hisG his1Δ::hisG/his1Δ::hisG* | Auxotrophic wild type | [[2](#_ENREF_2)] |
| BWP17 + CIp30 | *ura3Δ:: λimm434/ura3Δ::λimm434 arg4Δ::hisG/arg4Δ::hisG his1Δ::hisG/his1Δ::hisG RPS10/rps10Δ::CIp30-URA3-HIS1-ARG4* | Wild type | [[3](#_ENREF_3)] |
| CAI-4 | *ura3Δ::λimm434/ura3Δ::λimm434* | Auxotrophic wild type | [[4](#_ENREF_4)] |
| CAI-4 + CIp10  (NGY152) | *ura3Δ::λimm434/ura3Δ::λimm434 RPS10/rps10Δ::CIp10-URA3* | Wild type | [[5](#_ENREF_5)] |
| *pGFP* | *ura3Δ::λimm434/ura3Δ::λimm434 RPS10/rps10Δ::GFP-URA3* | GFP reporter | [[6](#_ENREF_6)] |
| *ACT1p-GFP* | *ura3Δ::λimm434/ura3Δ::λimm434 RPS10/rps10Δ::ACT1p-GFP-URA3* | GFP reporter | [[6](#_ENREF_6)] |
| *ICL1p-GFP* (CJB-3) | *ura3Δ::λimm434/ura3Δ::λimm434 RPS10/rps10Δ::ICL1p-GFP-URA3* | GFP reporter | [[7](#_ENREF_7)] |
| *CTA1p-GFP* | *ura3Δ::λimm434/ura3Δ::λimm434 RPS10/rps10Δ::CTA1p-GFP-URA3* | GFP reporter | [[8](#_ENREF_8)] |
| *MLS1p-GFP* | *ura3Δ::λimm434/ura3Δ::λimm434 RPS10/rps10Δ::MLS1p-GFP-URA3* | GFP reporter | This study |
| *PCK1p-GFP* (CJB-2) | *ura3Δ::λimm434/ura3Δ::λimm434 RPS10/rps10Δ::PCK1p-GFP-URA3* | GFP reporter | [[7](#_ENREF_7)] |
| *SOD5p-GFP* | *ura3Δ::λimm434/ura3Δ::λimm434 RPS10/rps10Δ::SOD5p-GFP-URA3* | GFP reporter | [[6](#_ENREF_6)] |
| *SSU1p-GFP* | *ura3Δ::λimm434/ura3Δ::λimm434 RPS10/rps10Δ::SSU1p-GFP-URA3* | GFP reporter | This study |
| *GRX2p-GFP*  (*TTR1p-GFP*) | *ura3Δ::λimm434/ura3Δ::λimm434 RPS10/rps10Δ::GRX2p-GFP-URA3* | GFP reporter | [[8](#_ENREF_8)] |
| *TRX1p-GFP* | *ura3Δ::λimm434/ura3Δ::λimm434 RPS10/rps10Δ::TRX1p-GFP-URA3* | GFP reporter | [[8](#_ENREF_8)] |
| *YHB1p-GFP* | *ura3Δ::λimm434/ura3Δ::λimm434 RPS10/rps10Δ::YHB1p-GFP-URA3* | GFP reporter | This study |
| *grx2Δ/Δ* + CIp10  (GCY207) | *ura3Δ::λimm434/ura3Δ::λimm434 grx2Δ::hisG/grx2Δ::hisG RPS10/rps10Δ::CIp10-URA3* | Glutathione reductase mutant | [[9](#_ENREF_9)] |
| cta1Δ/Δ  (C1-11) | *ura3Δ::imm434/ura3Δ::imm434 his1Δ::hisG/his1Δ::hisG cta1Δ::loxP-URA3-loxP/cta1Δ::HIS1* | Catalase mutant | 1. Brown (unpublished) |
| CA-IF100 | *arg4Δ/arg4Δ leu2Δ/leu2Δ::CmLEU2 his1Δ/his1Δ::CdHIS1 URA3/ura3Δ* | Wild type | [[10](#_ENREF_10)] |
| *sod1*Δ/Δ  (CA-IF003) | *arg4Δ/arg4Δ leu2Δ/leu2Δ his1Δ/his1Δ URA3/ura3Δ sod1Δ::CmLEU2/sod1Δ::CdHIS1* | Superoxide dismutase 1 mutant | [[10](#_ENREF_10)] |
| *sod4*Δ/Δ  (CA-IF015) | *arg4Δ/arg4Δ leu2Δ/leu2Δ his1Δ/his1Δ URA3/ura3Δ sod4Δ::CmLEU2/sod4Δ::CdHIS1* | Superoxide dismutase 4 mutant | [[10](#_ENREF_10)] |
| *sod5*Δ/Δ  (CA-IF019) | *arg4Δ/arg4Δ leu2Δ/leu2Δ his1Δ/his1Δ URA3/ura3Δ sod5Δ::CmLEU2/sod5Δ::CdHIS1* | Superoxide dismutase 5 mutant | [[10](#_ENREF_10)] |
| *sod5*Δ/Δ + *SOD5*  (CA-IF027) | *arg4Δ/arg4Δ leu2Δ/leu2Δ his1Δ/his1Δ URA3/ura3Δ sod5Δ::CmLEU2/sod5Δ::CdHIS1::SOD5-FRT* | Superoxide dismutase 5 reconstituted strain | [[10](#_ENREF_10)] |
| *sod6*Δ/Δ  (CA-IF023) | *arg4Δ/arg4Δ leu2Δ/leu2Δ his1Δ/his1Δ URA3/ura3Δ sod6Δ::CmLEU2/sod6Δ::CdHIS1* | Superoxide dismutase 6 mutant | [[10](#_ENREF_10)] |
| *sod4/5*Δ/Δ  (CA-IF039) | *arg4Δ/arg4Δ leu2Δ/leu2Δ his1Δ/his1Δ URA3/ura3Δ sod5Δ::CmLEU1/sod5Δ::CdHIS1 sod4Δ::FRT/sod4Δ::FRT* | Superoxide dismutase 4 and 5 double mutant | [[10](#_ENREF_10)] |
| *sod5Δ/Δ sod4Δ/Δ sod6Δ/Δ*  (CA-IF070) | *arg4/arg4 leu2/leu2 his1/his1 URA3/ura3 sod5Δ::CmLEU1/sod5Δ::CdHIS1 sod4Δ::FRT/sod4Δ::FRT sod6Δ::FRT/sod6Δ::FRT* | Superoxide dismutase 4, 5 and 6 triple mutant | [[10](#_ENREF_10)] |
| *hog1Δ/Δ cap1Δ/Δ*  (JC118) | *ura3Δ::λimm434/ura3Δ::λimm434 his1Δ::hisG/his1Δ::hisG arg4Δ::hisG/arg4Δ::hisG hog1Δ::loxP-ARG4-ura3Δ-loxP/hog1Δ::loxP-HIS1-loxP cap1Δ::hisG/cap1Δ::hisG-URA3-hisG* | Hog1 and Cap1 double mutant | [[11](#_ENREF_11)] |
| SN148 + CIp30  (JC747) | *arg4Δ/arg4Δ leu2Δ/leu2 his1Δ/his1Δ ura3Δ::λimm434/ura3::λimm434*  *iro1Δ::λimm434/iro1Δ::λimm434 RPS10/rps10Δ::CIp30-URA3-HIS1-ARG4* | Wild type | [[12](#_ENREF_12)] |
| *trx1Δ/Δ* + CIp10 | *arg4Δ/arg4Δ leu2Δ/leu2 his1Δ/his1Δ ura3Δ::λimm434/ura3::λimm434*  *iro1Δ::λimm434/iro1Δ::λimm434 trx1Δ::loxP-ARG4-loxP/trx1Δ::loxP-HIS1-loxP RPS10/rps10Δ::CIp10-URA3* | Thioredoxin mutant | [[12](#_ENREF_12)] |
| RM1000 + CIp20  (CLM19-3) | *ura3Δ::λ imm434/ura3Δ::λ imm434 his1Δ::hisG/his1Δ::hisG RPS10/rps10Δ::CIp20-URA3-HIS1* | Wild type | [[7](#_ENREF_7)] |
| *icl1Δ/Δ* + CIp20  (CLM25-5) | *ura3Δ::λ imm434/ura3Δ::λ imm434 his1Δ::hisG/his1Δ::hisG icl1Δ::HIS1/icl1Δ::ura3 RPS10/rps10Δ::CIp20-URA3-HIS1* | Isocitrate lyase mutant | [[7](#_ENREF_7)] |
| *pck1Δ/Δ* + CIp20  (CLM56-4) | *ura3Δ::λ imm434/ura3Δ::λ imm434 his1Δ::hisG/his1Δ::hisG pck1Δ::HIS1/pck1Δ::ura3 RPS10/rps10Δ::CIp20-URA3-HIS1* | Phosphoenolpyruvate carboxykinase mutant | [[7](#_ENREF_7)] |
| *hog1Δ +* CIp20 | *ura3Δ::λimm434/ura3Δ::λimm434, his1Δ::hisG/his1Δ::hisG hog1Δ::loxP-ura3-loxP/hog1Δ::loxP-HIS1-loxP CIp20-URA3-HIS* | Hog1 mutant | [[13](#_ENREF_13)] |
| BH117 | *ura3::URA3/ura3Δ:: imm434, his1::HIS1/his1Δ::hisG, iro1::IRO1/iro1Δ::imm434* | Wild type | [[14](#_ENREF_14)] |
| yhb1Δ/Δ  (BH79) | *ura3::URA3/ura3Δ:: imm434, his1Δ::HisG/his1Δ::HisG, iro1::IRO1/iro1Δ:: imm434 yhb1Δ::HisG/yhb1Δ::HIS1* | Nitric oxide dioxygenase Yhb1 mutant | [[14](#_ENREF_14)] |
| *ssu1Δ/Δ* | *ssu1Δ::FRT/ssu1Δ::FRT* | Sulphite transport protein mutant | P. Staib (unpublished) |

1. Gillum AM, Tsay EY, Kirsch DR (1984) Isolation of the *Candida albicans* gene for orotidine-5'-phosphate decarboxylase by complementation of *S. cerevisiae* ura3 and *E. coli* pyrF mutations. Mol Gen Genet 198: 179-182.

2. Wilson RB, Davis D, Mitchell AP (1999) Rapid hypothesis testing with *Candida albicans* through gene disruption with short homology regions. J Bacteriol 181: 1868-1874.

3. Zakikhany K, Naglik JR, Schmidt-Westhausen A, Holland G, Schaller M, et al. (2007) In vivo transcript profiling of *Candida albicans* identifies a gene essential for interepithelial dissemination. Cell Microbiol 9: 2938-2954.

4. Fonzi WA, Irwin MY (1993) Isogenic strain construction and gene mapping in *Candida albicans*. Genetics 134: 717-728.

5. Brand A, MacCallum DM, Brown AJ, Gow NA, Odds FC (2004) Ectopic expression of *URA3* can influence the virulence phenotypes and proteome of *Candida albicans* but can be overcome by targeted reintegration of *URA3* at the *RPS10* locus. Eukaryot Cell 3: 900-909.

6. Fradin C, De Groot P, MacCallum D, Schaller M, Klis F, et al. (2005) Granulocytes govern the transcriptional response, morphology and proliferation of *Candida albicans* in human blood. Mol Microbiol 56: 397-415.

7. Barelle CJ, Priest CL, Maccallum DM, Gow NA, Odds FC, et al. (2006) Niche-specific regulation of central metabolic pathways in a fungal pathogen. Cell Microbiol 8: 961-971.

8. Enjalbert B, MacCallum DM, Odds FC, Brown AJ (2007) Niche-specific activation of the oxidative stress response by the pathogenic fungus *Candida albicans*. Infect Immun 75: 2143-2151.

9. Chaves GM, Bates S, Maccallum DM, Odds FC (2007) *Candida albicans GRX2*, encoding a putative glutaredoxin, is required for virulence in a murine model. Genet Mol Res 6: 1051-1063.

10. Frohner IE, Bourgeois C, Yatsyk K, Majer O, Kuchler K (2008) *C. albicans* Cell Surface Superoxide Dismutases Degrade Host-Derived Reactive Oxygen Species to Escape Innate Immune Surveillance. Mol Microbiol.

11. Enjalbert B, Smith DA, Cornell MJ, Alam I, Nicholls S, et al. (2006) Role of the Hog1 stress-activated protein kinase in the global transcriptional response to stress in the fungal pathogen *Candida albicans*. Mol Biol Cell 17: 1018-1032.

12. da Silva Dantas A, Patterson MJ, Smith DA, Maccallum DM, Erwig LP, et al. (2010) Thioredoxin regulates multiple hydrogen peroxide-induced signaling pathways in *Candida albicans*. Mol Cell Biol 30: 4550-4563.

13. Smith DA, Nicholls S, Morgan BA, Brown AJ, Quinn J (2004) A conserved stress-activated protein kinase regulates a core stress response in the human pathogen *Candida albicans*. Mol Biol Cell 15: 4179-4190.

14. Hromatka BS, Noble SM, Johnson AD (2005) Transcriptional response of *Candida albicans* to nitric oxide and the role of the *YHB1* gene in nitrosative stress and virulence. Mol Biol Cell 16: 4814-4826.
