# Supplementary material for: Cellular Responses of Candida albicans to Phagocytosis and the Extracellular Activities of Neutrophils Are Critical to Counteract Carbohydrate Starvation, Oxidative and Nitrosative Stress
Source: PLoS One. 2012 Dec 21;7(12):e52850. doi: 10.1371/journal.pone.0052850 (PMC3528649; doi:10.1371/journal.pone.0052850)
Supplement: Table S2 — Primers used in this work. (DOCX) [file pone.0052850.s004.docx]

**Table S2.** Primers used in this work.

| **Primer** | **Sequence (5’ – 3’)** | **Reference** |
| --- | --- | --- |
| MLS1 Xho1 5’ | ctcgagatagatttttatttattctgatctga | This study |
| MLS1 HindIII 3’ | aagctttatgtaacttggttaatatatatacaaagc | This study |
| SSU1 XhoI 5’ | ctcgagaatatatcttgaaaccaagaggaaaatc | This study |
| SSU1 PstI 3’ | ctgcagtattgcaaaaacaagaagaacaag | This study |
| YHB1 XhoI 5’ | ctcgagaactacttcaaaacgtcacca | This study |
| YHB1 MluI 3’ | acgcgtgtttagctgctctgttctaaag | This study |
